# Systematic review and meta-analysis of specific external Chinese herbal medicines for post-stroke dysphagia: efficacy and clinical implications

**DOI:** 10.3389/fphar.2025.1635090

**Published:** 2025-09-01

**Authors:** Jingwen Zhang, Xingyu Kang, Jingwen Zhao, Jiajie Niu, Siyu Chen, Shuai Shi

**Affiliations:** ^1^ Second Clinical Medical College, Heilongjiang University of Traditional Chinese Medicine, Harbin, Heilongjiang, China; ^2^ Department of Geriatrics II, The Second Affiliated Hospital of Heilongjiang University of Traditional Chinese Medicine, Harbin, Heilongjiang, China

**Keywords:** deglutition disorders, external traditional Chinese medicine therapies, stroke, therapeutic efficacy, systematic review

## Abstract

**Objective:**

To systematically evaluate the efficacy and safety of specific external traditional Chinese medicine therapies (SETCM therapies) versus conventional non-SETCM therapy interventions for improving swallowing function, nutritional status, and reducing complications in adult patients with post-stroke dysphagia (PSD), based on randomized controlled trials (RCTs).

**Methods:**

A comprehensive search was conducted from inception to present across Chinese [(China National Knowledge Infrastructure (CNKI), VIP Chinese Science and Technology Periodical Database (VIP), Wanfang Data Knowledge Service Platform (WFSD), Chinese Biology Medicine Database (CBM)] and international databases (PubMed, Embase, Web of Science, and Cochrane Library). RCTs investigating validated SETCM therapy modalities (e.g., herbal patches, iontophoresis, compresses, and fumigation) for PSD were included. All herbal components were taxonomically validated using the Kew Medicinal Plant Names Service (MPNS). The control group received conventional therapy (rehabilitation, nursing, and medication). The experimental group received SETCM therapies alone or combined with conventional care. The risk of bias in eligible studies was assessed using the Cochrane risk-of-bias tool. A meta-analysis was performed using RevMan 5.4. Stratified subgroup analyses were conducted based on stroke lesion location (supratentorial vs. brainstem) and intervention type.

**Results:**

Twenty-five RCTs (n = 2,159 patients; SETCM therapies group = 1,152, control group = 1,007) were included. The meta-analysis demonstrated significantly greater benefits in the SETCM therapies group for: overall response rate (OR = 3.28, 95% CI [2.49, 4.31]); overall cure rate (OR = 2.36, 95% CI [1.84, 3.02]); water swallowing test (WST) score (MD = −0.65, 95% CI [−1.23, −0.06]); and SWAL-quality-of-life (SWAL-QOL) score (MD = 25.61, 95% CI [20.54, 30.67]). The SETCM therapies group also demonstrated superior results in videofluoroscopic swallow study (VFSS) scores, modified Barthel index (MBI), serum albumin (ALB), prealbumin (PA), gugging swallowing screen (GUSS), activities of daily living (ADL), and functional dysphagia scale (FDS) scores, nasogastric tube removal success rate, as well as lower Nutritional Risk Screening 2002 (NRS 2002) scores, reduced aspiration incidence, and shorter nasogastric tube indwelling time. Safety analysis (three studies) reported mild skin irritation (erythema and pruritus) in 2.1% of cases.

**Conclusion:**

SETCM therapies significantly improved swallowing function, nutritional status, and clinical outcomes in patients with PSD. Efficacy exhibited neuroanatomical specificity: Acupoints are preferred for cerebral hemisphere lesions, while herbal iontophoresis is the best choice for brainstem involvement. These findings support the integration of targeted SETCM therapies into PSD rehabilitation. However, the evidence is limited by methodological biases, necessitating high-quality RCTs for confirmation.

**Systematic Review Registration:**

https://www.crd.york.ac.uk/PROSPERO/, identifier CRD42024599344.

## 1 Introduction

Stroke, a prevalent neurological disorder resulting from the narrowing, obstruction, or rupture of cerebral blood vessels, is characterized by high morbidity and severe sequelae, significantly impairing patients’ quality of life (Dou, 2009). Among its complications, post-stroke dysphagia (PSD) ranks as one of the most frequent (Martino et al., 2005), with an incidence rate as high as 81% (Alamer et al., 2020). Crucially, a history of stroke is a well-established risk factor for dysphagia (Mourão et al., 2016). Although approximately 60% of patients regain partial swallowing function within 3 months, 11%–50% exhibit persistent dysfunction at 6 months (Song et al., 2024; Xiao et al., 2020). This dysfunction is closely associated with impaired respiratory function (Song et al., 2024; Xiao et al., 2020), increasing the risk of aspiration pneumonia by 3.8-fold and mortality by 11%–16% (Umay et al., 2022; Chang et al., 2022), thereby hindering rehabilitation (Han et al., 2020).

Current modern medical treatments for PSD primarily include oral sensory stimulation training, oral motor exercises, and physical therapy (Hui and Zhang, 2025). Traditional Chinese medicine (TCM) has accumulated extensive experience in managing PSD, achieving favorable outcomes through methods such as swallowing rehabilitation training, oral herbal medicine administration, and acupoint pressure/massage. Although diverse therapeutic approaches for dysphagia exist, the clinical effectiveness of their individual or combined application remains unclear (Yang and Do, 2022). Furthermore, the high cost of treatment imposes a substantial burden on families and the healthcare system (Qureshi et al., 2022).

Consequently, identifying an economical, convenient, effective, and clinically feasible treatment modality capable of enhancing swallowing ability, improving nutritional intake, reducing complications, and facilitating recovery in PSD patients holds significant practical importance. In recent years, guided by TCM meridian theory and the concepts of Qi and blood, and integrated with modern medical technology, research exploring the mechanisms of transdermal drug delivery via acupoints has gained momentum. Specific external traditional Chinese medicine therapies (SETCM therapies), recognized as noninvasive and safe interventions, have consequently garnered increasing attention, offering novel perspectives for PSD rehabilitation (Zheng et al., 2024; Li, 2023). SETCM therapies refer to standardized interventions using pharmacopeia-defined herbal formulations applied via specific modalities (e.g., patching and iontophoresis) at anatomically targeted sites. High-quality randomized controlled trials (RCTs) investigating SETCM therapies for PSD have emerged, progressively focusing on evaluating their efficacy. However, this specific dimension has not been adequately explored in previous meta-analyses, highlighting an urgent need to synthesize the latest evidence using evidence-based medicine (EBM) methodologies.

Therefore, this systematic review assessed RCTs comparing SETCM therapy interventions with non-SETCM therapy controls in adult PSD patients, with a focus on swallowing function, nutritional status, and complications. The aims were to provide more reliable EBM evidence for clinical practice, explore the potential clinical utility of SETCM therapies in PSD management, and thereby offer valuable references for clinicians and rehabilitation therapists.

## 2 Materials and methods

### 2.1 Study registration

This study adhered to international guidelines for conducting and reporting meta-analyses concerning the selection and utilization of research methodologies. The study protocol was prospectively registered on the PROSPERO international prospective register of systematic reviews (https://www.crd.york.ac.uk/PROSPERO/), registration number CRD42024599344.

### 2.2 Literature search strategy

A comprehensive search was conducted across eight electronic databases. Chinese literature databases included China National Knowledge Infrastructure (CNKI), China Biology Medicine disc (SinoMed/CBM), VIP Chinese Science and Technology Periodical Database (VIP), and Wanfang Data Knowledge Service Platform (WFSD). International literature databases included PubMed, Embase, Web of Science, and the Cochrane Library. The search aimed to identify randomized controlled trials (RCTs) investigating specific external traditional Chinese medicine (SETCM) therapy interventions for post-stroke dysphagia (PSD). The search timeframe generally spanned from the inception of each database to the latest available date to ensure the inclusion of the most recent research. Search terms incorporated both medical subject headings (MeSH) and free-text words in Chinese and English: Dysphagia: (“Dysphagia” OR “Swallowing Disorders” OR “Deglutition Disorders” OR “Pseudobulbar Palsy” OR “Oropharyngeal Dysfunction”) Stroke: (“Stroke” OR “Cerebrovascular Accident” OR “Brain Infarction” OR “Cerebral Embolism” OR “Cerebral Ischemia”) SETCM therapy interventions: (“External Traditional Chinese Medicine” OR “Chinese Herbal Patch” OR “Iontophoresis” OR “Hot Compress” OR “Cold Compress” OR “Fumigation” OR “Herbal Steam” OR “Herbal Wash”).

Search strategies utilized Boolean operators (AND, OR), wildcards, and proximity operators where appropriate, combining MeSH terms and free-text keywords. Manual searches of relevant specialized journals and efforts to locate unpublished studies (grey literature) were also performed when necessary.

### 2.3 Definition, component standardization, and taxonomic validation of SETCM therapy interventions

SETCM therapies are standardized interventions using pharmacopeia-defined herbal formulations applied via specific methods (e.g., patching and iontophoresis) to anatomically defined target sites (typically acupoints). For all included primary studies, detailed information regarding the specific SETCM therapy interventions (including, but not limited to, herbal patching, iontophoresis, fumigation, and medicated stick application) was systematically extracted and re-examined. This encompassed the botanical components, preparation methods, and quality control standards (where mentioned). The botanical names of all reported medicinal plant species were subjected to systematic taxonomic validation using authoritative international databases (Rivera et al., 2014). The Consensus on Phytovigilance for Medicinal Plants (ConPhyMP) tool was employed to evaluate all included studies (Heinrich et al., 2018). This assessment covered: botanical names (scientific name, family, and genus), the plant parts used, functional components, pharmacological effects, mechanism of action, etc.

### 2.4 Literature inclusion and exclusion criteria

#### 2.4.1 Inclusion criteria

#### 2.4.2 Exclusion criteria

Studies were excluded based on the following criteria:

(1) Dysphagia caused by other underlying conditions (e.g., neurological disorders, head/neck cancer, or congenital abnormalities);
(2) Animal studies, case reports, conference abstracts, reviews, or non-research articles;
(3) Studies where the full text was unavailable or original data were incomplete/unrecoverable;
(4) Studies with major methodological flaws (e.g., lack of randomization and absence of a control group) or meeting any of the following conditions:

a. “High risk” of bias: Studies rated as “high risk” in ≥2 key domains (random sequence generation, allocation concealment, and blinding) using the Cochrane risk-of-bias tool;

b. Insufficient data integrity: Missing data for critical outcome measures where authors could not be contacted for clarification.

(Note: Studies rated as “unclear risk” in only one domain (e.g., allocation concealment) were retained due to the current limited availability of high-quality RCTs in the PSD field. The potential influence of these studies on conclusions was assessed via sensitivity analysis.)

(5) Patients with psychiatric disorders or severe communication barriers affecting informed consent;
(6) Studies lacking predefined primary/secondary outcomes or employing non-standardized assessment tools;
(7) Studies involving duplicate publication or overlapping datasets with prior publications.

### 2.5 Literature screening and data extraction

Literature screening: Two independent researchers performed the literature search and initial screening based on the predefined search strategy. EndNote 20 was used for reference management (including import, organization, merging, and deduplication). Initial screening involved excluding clearly irrelevant records based on titles and abstracts. Full texts of potentially relevant studies were then retrieved and scrutinized to determine eligibility. Attempts were made to contact authors for missing data from potentially eligible studies; studies were excluded if no response was received. Results from both initial and full-text screening underwent cross-checking by both researchers. Disagreements were resolved through discussion or arbitration by a third reviewer.

Data extraction: Extracted data encompassed: (1) basic study information (authors, year, and journal); (2) patient baseline characteristics (sample size, age, gender, stroke type/location, and time since stroke); (3) intervention details (SETCM therapy type, specific herbs/formulations [validated taxonomically], preparation, dosage, frequency, duration, and concurrent therapies); (4) control intervention details; (5) outcome measures (primary and secondary, as defined in Table 1, including measurement time points and scales used); and (6) quality assessment data. To standardize efficacy assessment, the end of the intervention period was selected as the primary observation endpoint. Continuous outcome data were recorded as mean ± standard deviation ( $\bar{x}$ 7± s). Missing data were requested from the original authors; if unavailable, they were documented as missing.

**TABLE 1:** PICOS strategy—inclusion criteria.

PICOS	Inclusion criteria
Population	Adult patients (>18 years old) diagnosed with post-stroke dysphagia (PSD) according to established diagnostic criteria (e.g., criteria from the 4th National Cerebrovascular Disease Conference), with stroke confirmed by CT or MRI
Intervention	Experimental group receiving validated external traditional Chinese medicine therapies (SETCM therapies) alone (e.g., herbal patching, TCM iontophoresis, hot compress, fumigation, fumigation-wash, wash-soak, herbal hot compress, cold compress) or SETCM therapies combined with conventional care
Comparison	Control group receiving non-SETCM therapy interventions: conventional therapy (e.g., medication), conventional nursing care, or conventional rehabilitation training
Outcomes	Primary outcomes: Overall response rate (defined as the percentage of cases achieving comprehensive cure, marked improvement, or improvement); Cure rate (defined as the percentage of cases achieving cure status); Water swallowing test (WST) score; Swallow quality-of-life questionnaire (SWAL-QOL) score; Complication incidence rate. Secondary outcomes: Videofluoroscopic swallow study (VFSS) score; Modified Barthel index (MBI) score; Gugging swallowing screen (GUSS) score; Activities of daily living (ADL) score; Nasogastric tube indwelling duration; Feeding tube removal success rate; Nutritional risk screening 2002 (NRS 2002) score; Fujishima dysphagia scale (FDS) score; Nutritional biomarkers: Serum albumin (ALB), Prealbumin (PA); Safety data: Incidence of skin reactions/systemic adverse events
Study design	Randomized controlled trials (RCTs)

Note: P: population; I: intervention; C: comparison; O: outcome; S: study design; CT: computed tomography; MRI: magnetic resonance imaging.

### 2.6 Assessment of methodological quality (risk of bias)

The methodological quality (risk of bias) of included randomized controlled trials (RCTs) was assessed independently by two reviewers using the Cochrane risk-of-bias tool (RoB 2) for randomized trials. The assessment comprised two parts. In the Descriptive assessment, Detailed descriptions of study methodology relevant to each bias domain were recorded to support the judgment of risk. In the Judgment of Risk assessment, each domain was judged as “Low risk,” “High risk,” or “Unclear risk” of bias.

The specific bias domains evaluated were: Selection Bias: Random sequence generation, Allocation concealment; Performance Bias: Blinding of participants and personnel; Detection Bias: Blinding of outcome assessment; Attrition Bias: Incomplete outcome data; Reporting Bias: Selective outcome reporting; Other Bias: Any other potential sources of bias not covered above.

Disagreements in judgments were resolved through consensus discussion or consultation with a third reviewer.

### 2.7 Statistical analysis

Statistical analyses of the extracted outcome data were performed using Review Manager (RevMan) software, version 5.4.1, provided by the Cochrane Collaboration. Heterogeneity among studies for each clinical outcome was assessed using the chi^2^ test (Q statistic) and quantified using the I^2^ statistic. A significance level of *P* > 0.10 for the chi^2^ test indicated acceptable heterogeneity, warranting the use of a fixed-effects model (FEM) for meta-analysis. Conversely, a chi^2^ test result of *P* ≤ 0.10 indicated significant heterogeneity, leading to the use of a random-effects model (REM) for meta-analysis. The magnitude of heterogeneity was interpreted based on I^2^ values: I^2^ ≤ 25% indicated low heterogeneity, I^2^ > 50% indicated moderate heterogeneity, and I^2^ > 75% indicated substantial heterogeneity. Where significant heterogeneity was present, sensitivity analyses were conducted using the study removal method to identify potential sources and exclude studies contributing excessively to heterogeneity. For continuous outcomes, results were pooled using the standardized mean difference (SMD) with 95% confidence intervals (CI). For dichotomous outcomes, the relative risk (RR) with 95% CI was used. Results were presented using forest plots. Publication bias was assessed visually using inverted funnel plots plotting the RR (for dichotomous outcomes) or SMD (for continuous outcomes) against its standard error (SE). Two predefined subgroup analyses were performed: (a) stratified by SETCM therapy intervention type (herbal patching, TCM iontophoresis, medicated stick application); and (b) stratified by stroke lesion location (supratentorial [cerebral hemisphere] vs. brainstem). Studies not reporting specific lesion location were excluded from the latter subgroup analysis.

### 2.8 GRADE assessment of evidence quality

Two independent researchers assessed the quality of evidence (certainty) for each outcome measure using the Grading of Recommendations Assessment, Development and Evaluation (GRADE) approach (Guyatt et al., 2011). Assessments were conducted using the online GRADEpro Guideline Development Tool (GRADEpro GDT; https://gradepro.org). The quality of evidence for each outcome was downgraded based on five domains: Risk of bias across included studies; Inconsistency: Unexplained heterogeneity in results; Indirectness: Indirectness of population, intervention, comparator, or outcome; Imprecision: Wide confidence intervals or small sample size; Publication bias: Evidence of publication bias. The initial quality rating for RCTs is “High.” Evidence quality was then categorized as High: No downgrading (we are very confident that the true effect lies close to that of the estimate); Moderate: Downgraded by one level (we are moderately confident in the effect estimate; the true effect is likely close to the estimate, but there is a possibility of being substantially different); or Low: Downgraded by two levels (our confidence in the effect estimate is limited; the true effect may be substantially different from the estimate). Very low: Downgraded by three levels (we have very little confidence in the effect estimate; the true effect is likely substantially different from the estimate).

Disagreements between reviewers were resolved through discussion or arbitration by a third reviewer.

## 3 Results

### 3.1 Literature screening results and characteristics of included studies

**FIGURE 1:**
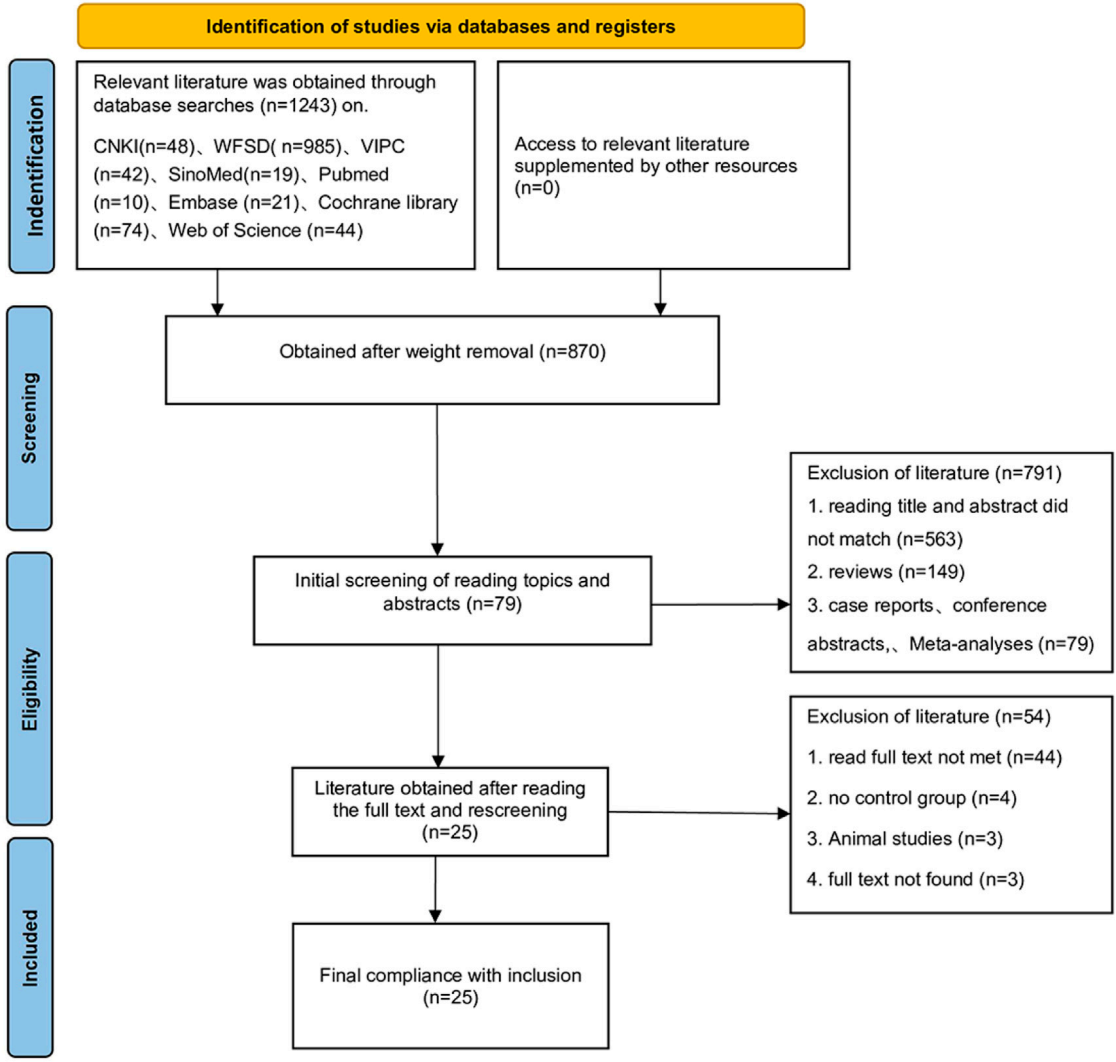
PRISMA flow chart.

The initial search identified 1,243 potentially relevant records. After removing 373 duplicate publications, 870 unique records remained. Screening of titles and abstracts excluded 791 records, leaving 79 articles for full-text assessment. Following full-text review, 54 articles were excluded, resulting in the final inclusion of 25 published randomized controlled trials (RCTs). The study selection process is detailed in Figure 1. The baseline characteristics of the included studies are summarized in Table 2. These 25 RCTs enrolled a total of 2,245 participants. Regarding the specific SETCM therapy modalities employed: 16 studies utilized acupoint herbal patching, two studies utilized herbal fumigation, two studies utilized TCM iontophoresis, two studies utilized medicated herbal stick application, and two studies utilized acupoint stimulation with herbal ice-cotton swabs. One study utilized targeted drug delivery therapy. Concerning the intervention durations: 15 studies implemented an intervention period of approximately 4 weeks, six studies implemented an intervention period of 3 weeks, and four studies implemented an intervention period of 2 weeks. The results of the included studies indicated that there were no significant differences in baseline measure scores between the experimental (SETCM therapies) and control groups across all studies (*P* > 0.05). Post-intervention, outcome measure scores improved significantly compared to baseline within both groups (*P* < 0.05). The improvement observed in the experimental (SETCM therapies) group was significantly greater than that in the control group (*P* < 0.05).

**TABLE 2:** Baseline characteristics of included studies and details of SETCM therapy interventions.

Author(s)	Year	Sample size	Age	Days	Outcome indicator	Intervention measures			Stroke location	Herbal medicine used
						T	C	Operational area		
Tang and Liu (2024)	2024	34/34	64.18 ± 8.09/64.28 ± 8.14	30 days	⑥⑪ ⑫ ⑬ ⑭	Acupoint application + rehabilitation	Rehabilitation	Tiantu point, Renying point, and Lianquan point	Unspecified	*Asari Radix et Rhizoma, Aconiti Lateralis Radix Praeparata, Pinelliae Rhizoma, Arisaematis Rhizoma Preparatum, Borneolum Syntheticum, Fritillariae Cirrhosae Bulbus*
Yu and Wu (2024)	2024	75/75	45–78/42–80	28 days	①②④⑤	Acupoint application + rehabilitation	Rehabilitation	Tiantu point, Renying point, and Lianquan point	Supratentorial	*Pinelliae Rhizoma Praeparatum,Acori Tatarinowii Rhizoma, Arisaematis Rhizoma Preparatum, Notoginseng Radix et Rhizoma, Aconiti Lateralis Radix Praeparata, Asari Radix et Rhizoma*
Wang and Sun (2023)	2023	15/15	58–82/59–80	28 days	③⑤⑦⑧⑨	Acupoint application + rehabilitation	Rehabilitation	Tiantu point, Renying point, and Lianquan point	Unspecified	*Pinelliae Rhizoma, Arisaematis Rhizoma Preparatum, Aconiti Lateralis Radix Praeparata, Asari Radix et Rhizoma, Fritillariae Cirrhosae Bulbus, Borneolum Syntheticum*
Zhu et al. (2023)	2023	32/32	77.72 ± 7.87/80.75 ± 6.77	21 days	①②	targeted transdermal medication+ rehabilitation	Rehabilitation	Dazhui point and Shenshu point	Supratentorial	*Rehmanniae Radix Praeparata, Morindae Officinalis Radix, Corni Fructus, Dendrobii Caulis, Cistanches Herba, Aconiti Lateralis Radix Praeparata, Schisandrae Chinensis Fructus, Cinnamomi Cortex, Poria, Ophiopogonis Radix, Acori Tatarinowii Rhizoma, Polygalae Radix*
Yu and Qiu (2023)	2023	40/40	54–77/53–77	30 days	⑥⑪ ⑬ ⑭	Acupoint application + rehabilitation	Rehabilitation	Acupoint application + rehabilitation	Unspecified	*Asari Radix et Rhizoma, Aconiti Lateralis Radix Praeparata, Pinelliae Rhizoma, Arisaematis Rhizoma Preparatum, Borneolum Syntheticum, Fritillariae Cirrhosae Bulbus*
Wang et al. (2023b)	2023	50/50	20–75/20–75	14 days	①②③④	Acupoint application + medication	Medication	Renying point, Lianquan point, and Fengfu point	Supratentorial	*Persicae Semen, Carthami Flos, Angelicae Sinensis Radix, Paeoniae Radix Rubra, Rehmanniae Radix, Scrophulariae Radix, Platycodonis Radix, Aurantii Fructus, Borneolum Syntheticum, Glycyrrhizae Radix et Rhizoma*
Xu (2022)	2022	60/60	45–93/46–93	30 days	⑪ ⑬ ⑭	Acupoint application + rehabilitation	Rehabilitation	Tiantu point, Renying point, and Lianquan point	Unspecified	*Asari Radix et Rhizoma, Aconiti Lateralis Radix Praeparata, Pinelliae Rhizoma, Arisaematis Rhizoma Preparatum, Borneolum Syntheticum, Fritillariae Cirrhosae Bulbus*
Shi et al. (2022)	2022	66/66	46–74/43–73	30 days	①②⑫	Herbal fumigation + rehabilitation	Rehabilitation	neck	Unspecified	*Cinnamomi Cortex, Glycyrrhizae Radix et Rhizoma, Poria, Ginseng Radix et Rhizoma, Achyranthis Bidentatae Radix, Eucommiae Cortex, Rehmanniae Radix, Chuanxiong Rhizoma, Paeoniae Radix Rubra, Angelicae Sinensis Radix, Asari Radix et Rhizoma, Saposhnikoviae Radix, Gentianae Macrophyllae Radix, Taxilli Herba*
Zhou et al. (2022)	2022	75/75	33–69/32–65	21 days	①②③④	Acupoint application + conventional treatment+ rehabilitation	Conventional treatment + rehabilitation	Renying point, Lianquan point, and Fengfu point	Supratentorial	*Persicae Semen, Carthami Flos, Angelicae Sinensis Radix, Paeoniae Radix Rubra, Rehmanniae Radix, Scrophulariae Radix, Platycodonis Radix, Aurantii Fructus, Borneolum Syntheticum, Glycyrrhizae Radix et Rhizoma*
Jiao (2022)	2022	40/40	53–73/52–74	28 days	③⑤⑥	Acupoint application + conventional treatment	Conventional treatment	Tiantu point, Renying point, and Lianquan point	Unspecified	*Asari Radix et Rhizoma, Aconiti Lateralis Radix Praeparata, Pinelliae Rhizoma, Arisaematis Rhizoma Preparatum, Borneolum Syntheticum, Fritillariae Cirrhosae Bulbus*
Wu et al. (2021)	2021	40/40	47–83/46–81	30 days	①⑧⑩	Acupoint application + rehabilitation	Rehabilitation	Tiantu point, Renying point, and Lianquan point	Unspecified	*Asari Radix et Rhizoma, Aconiti Lateralis Radix Praeparata, Pinelliae Rhizoma, Arisaematis Rhizoma Preparatum, Borneolum Syntheticum, Fritillariae Cirrhosae Bulbus*
Dong et al. (2021)	2021	46/46	52–78/51–79	28 days	①⑧⑨	Acupoint application + conventional treatment+ rehabilitation	Conventional treatment +rehabilitation	Tiantu point, Renying point, and Lianquan point	Unspecified	*Asari Radix et Rhizoma, Aconiti Lateralis Radix Praeparata, Pinelliae Rhizoma, Arisaematis Rhizoma Preparatum, Borneolum Syntheticum, Fritillariae Cirrhosae Bulbus*
Shao and Chen (2020)	2020	53/53	40–79/41–77	28 days	①②③④⑪	Herbal medicine stick treatment + conventional treatment	Conventional treatment	Posterior palatal arch, soft palate, pharyngeal palatal arch, posterior pharyngeal wall, and root of tongue	Supratentorial	*Moschus, Menthae Haplocalycis Herba, Styrax, Borneolum Syntheticum*
Zheng et al. (2019)	2019	42/42	62.6 ± 7.5/63.4 ± 7.9	14 days	①②③	Acupoint application + electrical stimulation+ rehabilitation	Electrical stimulation + rehabilitation	Tiantu point, Renying point, Lianquan point, and Futu point	Supratentorial	*Moschus, Ginseng Radix et Rhizoma, Styrax, Bovis Calculus, Bufonis Venenum, Cinnamomi Cortex, Borneolum Syntheticum*
Hu (2019)	2019	61/61	65.3 ± 12.2/65.3 ± 12.2	30 days	①②③④⑤⑦⑧⑨⑩	Acupoint application + conventional treatment	Conventional treatment	Tiantu point, Renying point, and Lianquan point	Unspecified	*Asari Radix et Rhizoma, Aconiti Lateralis Radix Praeparata, Pinelliae Rhizoma, Arisaematis Rhizoma Preparatum, Borneolum Syntheticum, Fritillariae Cirrhosae Bulbus*
Zhou et al. (2018)	2018	40/40	36–72/38–70	21 days	①③④	Herbal medicine stick treatment + conventional treatment	Conventional treatment	Herbal medicine stick treatment + conventional treatment	Brainstem	*Bombyx Batryticatus, Acori Tatarinowii Rhizoma, Arisaematis Rhizoma Preparatum, Borneolum Syntheticum, Moschus, liquor*
Luo et al. (2018)	2018	35/35	43–82/39–85	14 days	①②④	Acupoint application + rehabilitation	Rehabilitation	Lianquan point	Supratentorial	*Kansui Radix, Sinapis Semen, Ephedrae Herba, Asari Radix et Rhizoma*
Ma et al. (2021)	2018	55/55	64.3 ± 11.2/63.5 ± 11.4	20 days	①②③④⑤⑮	Acupoint application + conventional treatment +rehabilitation	Conventional treatment + rehabilitation	Lianquan point, Bilateral Gongxue points, and Bilateral Renying points	Unspecified	*Pinelliae Rhizoma, Arisaematis Rhizoma Preparatum, Aconiti Lateralis Radix Praeparata, Asari Radix et Rhizoma, Acori Tatarinowii Rhizoma, Notoginseng Radix et Rhizoma Pulvis*
Xu and Zhao (2016)	2016	33/33	35–77/34–75	28 days	①②	Herbal fumigation + rehabilitation	Rehabilitation	Neck	Unspecified	*Cinnamomi Cortex, Glycyrrhizae Radix et Rhizoma, Poria, Ginseng Radix et Rhizoma, Achyranthis Bidentatae Radix, Eucommiae Cortex, Rehmanniae Radix, Chuanxiong Rhizoma, Paeoniae Radix Rubra, Angelicae Sinensis Radix, Asari Radix et Rhizoma, Saposhnikoviae Radix, Gentianae Macrophyllae Radix, Taxilli Herba*
Que and Bian (2016)	2016	43/43	66.88 ± 12.33/65.93 ± 12.61	20 days	①②③	Herbal medicine stick Treatment + rehabilitation	Rehabilitation	Both palatal arches, posterior pharyngeal wall, posterior root of the tongue, Jinjin points, and Yuyi points	Mixed	*Pheretima, Carthami Flos, Liquidambaris Fructus, Menthae Haplocalycis Herba, Borneolum Syntheticum*
Lai (2015)	2015	50/50	44–75/45–72	30 days	①	Acupoint application + rehabilitation	Rehabilitation	Fengchi point, Hegu point, and Jinjin point	Unspecified	*Astragali Radix, Angelicae Sinensis Radix, Paeoniae Radix Rubra, Atractylodis Macrocephalae Rhizoma, Chuanxiong Rhizoma, Carthami Flos, Persicae Semen, Glycyrrhizae Radix et Rhizoma*
Lin et al. (2014)	2014	30/29	60.47 ± 8.07/61.10 ± 9.23	30 days	①②⑤	Herbal medicine stick treatment + medication+ rehabilitation	Medication + rehabilitation	Uvula, bilateral palatopharyngeal arches, soft palate, and posterior root of tongue	Brainstem	*Arisaema Praeparatum, Pinelliae Rhizoma, Citri Exocarpium Rubrum, Bambusae Caulis in Taenias, Persicae Semen, Aurantii Fructus Immaturus, Carthami Flos, Poria, Acori Tatarinowii Rhizoma, Glycyrrhizae Radix et Rhizoma*
Zhao et al. (2012)	2012	23/23	35–84/35–84	30 days	①⑮	Acupoint application + rehabilitation	Rehabilitation	Tiantu point, Renying point, and Lianquan point	Unspecified	*Asari Radix et Rhizoma, Aconiti Lateralis Radix Praeparata, Pinelliae Rhizoma, Arisaematis Rhizoma Preparatum, Borneolum Syntheticum, Fritillariae Cirrhosae Bulbus*
Zhao et al. (2008)	2008	35/35	48–69/50–68	20 days	①②	TCM iontophoresis + rehabilitation	Rehabilitation	Lianquan point, Tiantu point, Fengfu point, Fengchi point, Yifeng point, and Hegu point	Brainstem	*Moschus, Borneolum Syntheticum*
Huang (2007)	2007	50/50	45–74/40–75	15 days	④	TCM iontophoresis + medication	Medication	Fengfu point, Lianquan point	Brainstem	-

Notes:

Outcomes assessed: ① Overall response rate; ② Cure rate; ③ Water swallowing test (WST) score; ④ Videofluoroscopic swallow study (VFSS) score; ⑤ Swallow quality-of-life questionnaire (SWAL-QOL) score; ⑥ Post-intervention aspiration rate; ⑦ Modified Barthel index (MBI) score; ⑧ Serum albumin (ALB); ⑨ Prealbumin (PA); ⑩ Nutritional risk screening 2002 (NRS 2002) score; ⑪ Gugging swallowing screen (GUSS) score; ⑫ Activities of daily living (ADL) score; ⑬ Nasogastric tube indwelling duration; ⑭ Feeding tube removal success rate; ⑮ Fujishima dysphagia scale (FDS) score. (See main text for abbreviation definitions).

Stroke lesion location classification:

Supratentorial: Lesions involving cortical/subcortical structures.

Brainstem: Lesions involving medullary/pontine structures.

Mixed/Unspecified: Excluded from lesion location-based efficacy analysis.

### 3.2 Development of a standardized SETCM therapy intervention database

This study successfully established a standardized database for specific external traditional Chinese medicine therapy (SETCM therapy) interventions. Specifically, through authoritative databases (MPNS v7.3+, POWO) and the *Chinese Pharmacopoeia* (2020 Edition), rigorous taxonomic validation and name standardization were performed on all medicinal plant species reported in the primary studies, ensuring the accuracy of the botanical origins of the herbs used. These validation results have been compiled and summarized in Table 3: Standardized botanical information.

**TABLE 3:** Standardized botanical information table.

Medicinal Material (Chinese)	Latin name (ChP 2020)	Citrus	Specific Epithet	Named person's initials	Family	Part used
Bajitian	*Morindae Officinalis Radix*	*Morinda*	*officinalis*	How	Rubiaceae	Dried root (Radix)
Fuzi	*Aconiti Lateralis Radix Praeparata*	*Aconitum*	*carmichaelii*	Debx.	Ranunculaceae	Processed daughter root
Jiangcan	*Bombyx Batryticatus*	*Bombyx*	*mori*	Linnaeus	Bombycidae	Dried larva killed by Beauveria bassiana
Jiezi	*Sinapis Semen*	*Sinapis*	*alba*	L. (Carl Linnaeus)	Brassicaceae	Dried ripe seed (Semen)
Bingpian	*Borneolum Syntheticum*	*Not mentioned*	*Not mentioned*	Not mentioned	Synthetic	Crystalline product from Cinnamomum branches
Bohe	*Menthae Haplocalycis Herba*	*Mentha*	*haplocalyx*	Briq.	Lamiaceae	Dried aerial part (Herba)
Baizhu	*Atractylodis Macrocephalae Rhizoma*	*Atractylodes*	*macrocephala*	Koidz.	Asteraceae	Dried rhizome (Rhizoma)
Chuanxiong	*Chuanxiong Rhizoma*	*Ligusticum*	*chuanxiong*	Hort.	Apiaceae	Dried rhizome (Rhizoma)
Chuanbeimu	*Fritillariae Cirrhosae Bulbus*	*Fritillaria*	*cirrhosa*	D.Don	Liliaceae	Dried bulb (Bulbus)
Dannanxing	*Arisaema cum Bile*	*Not mentioned*	*Not mentioned*	Not mentioned	Araceae	Bile-processed tuber of Arisaema
Dilong	*Pheretima*	*Pheretima*	*aspergillum*	(E.Perrier)	Megascolecidae	Eviscerated dried body (Lumbricus)
Dihuang	*Rehmanniae Radix*	*Rehmannia*	*glutinosa*	Libosch.	Scrophulariaceae	Dried root tuber (Radix)
Duzhong	*Eucommiae Cortex*	*Eucommia*	*ulmoides*	Oliv.	Eucommiaceae	Dried bark (Cortex)
Fangfeng	*Saposhnikoviae Radix*	*Saposhnikovia*	*divaricata*	(Turcz.)Schischk.	Apiaceae	Dried root (Radix)
Fuling	*Poria*	*Poria*	*cocos*	(Schw.) Wolf	Polyporaceae	Dried sclerotium (Sclerotium)
Fuzi	*Aconiti Lateralis Radix Praeparata*	*Aconitumm*	*carmichaelii*	Debx.	Ranunculaceae	Dried daughter root (Radix Aconiti Lateralis)
Gansui	*Kansui Radix*	*Euphorbia*	*kansui*	T.N.Liou ex T.P.Wang	Euphorbiaceae	Dried root tuber (Radix)
Gancao	*Glycyrrhizae Radix et Rhizoma*	*Glycyrrhiza*	*uralensis*	Fisch.	Fabaceae	Dried root and rhizome (Radix et Rhizoma)
Honghua	*Carthami Flos*	*Carthamus*	*tinctorius*	L.(Carl Linnaeus)	Asteraceae	Dried flower (Flos)
Huangqi	*Astragali Radix*	*Astragalus*	*membranaceus*	(Fisch.) Bge.	Fabaceae	Dried root (Radix)
Jiegeng	*Platycodonis Radix*	*Platycodon*	*grandiflorum*	(Jacq.)A.DC.	Campanulaceae	Dried Root (Radix Platycodi)
Juhong	*Citri Exocarpium Rubrum*	*Citrus*	*reticulata*	Blanco	Rutaceae	Dried Pericarp (Pericarpium Citri Reticulatae)
Lulu tong	*Liquidambaris Fructus*	*Liquidambar*	*formosana*	Hance	Hamamelidaceae	Dried Resin (Resina Liquidambaris)
Mahuang	*Ephedrae Herba*	*Ephedra*	*sinica*	Stapf	Ephedraceae	Dried Herbaceous Stem (Herba Ephedrae)
Maidong	*Ophiopogonis Radix*	*Ophiopogon*	*japonicus*	(L. f.) Ker-Gawl.	Liliaceae	Dried Root Tuber (Radix Ophiopogonis)
Niuxi	*Achyranthis Bidentatae Radix*	*Achyranthes*	*bidentata*	Bl.	Amaranthaceae	Dried Root (Radix Achyranthis)
Qinjiao	*Gentianae Macrophyllae Radix*	*Gentiana*	*macrophylla*	Pall.	Gentianaceae	Dried Root (Radix Gentianae)
Renshen	*Ginseng Radix et Rhizoma*	*Panax*	*ginseng*	C.A.Mey.	Araliaceae	Dried Root (Radix Ginseng)
Roucongrong	*Cistanches Herba*	*Cistanche*	*deserticola*	Y.C.Ma	Orobanchaceae	Dried Fleshy Stem with Scales (Herba Cistanches)
Rougui	*Cinnamomi Cortex*	*Cinnamomum*	*cassia*	Presl	Lauraceae	Dried Bark (Cortex Cinnamomi)
Sanqi	*Notoginseng Radix et Rhizoma*	*Panax*	*notoginseng*	(Burk.) F. H. Chen	Araliaceae	Dried Root (Radix Notoginseng)
Sangjisheng	*Taxilli Herba*	*Taxillus*	*chinensis*	(DC.)Danser	Loranthaceae	Dried Stem with Leaves (Ramulus Taxilli)
Shanzhuyu	*Corni Fructus*	*Cornus*	*officinalis*	Sieb. et Zucc.	Cornaceae	Dried Flesh of Fruit (Fructus Corni)
Shichangpu	*Acori Tatarinowii Rhizoma*	*Acorus*	*tatarinowii*	Schott	Araceae	Dried Rhizome (Rhizoma Acori Tatarinowii)
Shihu	*Dendrobii Caulis*	*Dendrobium*	*nobile*	Lindl.	Orchidaceae	Dried Stem (Caulis Dendrobii)
Shexiang	*Moschus*	*Moschus*	*berezovskii*	Flerov	Cervidae	Dried Secretion from Scent Gland (Moschus)
Suhexiang	*Styrax*	*Liquidambar*	*orientalis*	Mill.	Hamamelidaceae	Resin from Trunk (Styrax Liquidambaris)
Taoren	*Persicae Semen*	*Prunus*	*persica*	(L.)Batsch	Rosaceae	Dried Ripe Seed (Semen Persicae)
Wuweizi	*Schisandrae Chinensis Fructus*	*Schisandra*	*chinensis*	(Turcz.)Baill.	Magnoliaceae	Dried Ripe Fruit (Fructus Schisandrae)
Xixin	*Asari Radix et Rhizoma*	*Asarum*	*heterotropoides*	Fr.Schmidt	Aristolochiaceae	Dried Root and Rhizome (Radix et Rhizoma Asari)
Xuanshen	*Scrophulariae Radix*	*Scrophularia*	*ningpoensis*	Hemsl.	Scrophulariaceae	Dried Root (Radix Scrophulariae)
Yuanzhi	*Polygalae Radix*	*Polygala*	*tenuifolia*	Willd.	Polygalaceae	Dried Root (Radix Polygalae)
Zhiqiao	*Aurantii Fructus*	*Citrus*	*aurantium*	L. (Carl Linnaeus)	Rutaceae	Dried Immature Whole Fruit (Fructus Immaturus Aurantii)
Zhishi	*Aurantii Fructus Immaturus*	*Citrus*	*aurantium*	L. (Carl Linnaeus)	Rutaceae	Dried Nearly Mature Halved Fruit (Fructus Aurantii)
Zhuru	*Bambusae Caulis in Taenias*	*Bambusa*	*tuldoides*	Munro	Poaceae	Shavings from Middle Stem Layer (Caulis Bambusae in Taeniam)
Danggui	*Angelicae Sinensis Radix*	*Angelica*	*sinensis*	(Oliv.) Diels	Apiaceae	Dried Root (Radix Angelicae Sinensis)
Chishao	*Paeoniae Radix Rubra*	*Paeonia*	*lactiflora*	Pall.	Ranunculaceae	Dried Root (Radix Paeoniae Alba)
Niuhuang	*Bovis Calculus*	*Bos*	*taurus domesticus*	Gmelin	Bovidae	Dried Gallstone (Calculus Bovis)
Chansu	*Bufonis Venenum*	*Bufo*	*bufo gargarizans*	Cantor	Bufonidae	Lyophilized Parotoid Gland Secretion (Venenum Bufonis)

The ConPhyMP tool for plant monitoring of medicinal plants was used to conduct a comprehensive assessment of all included studies and to formulate standardized intervention measures. This assessment covered key elements, including botanical names (scientific name, family, and genus), plant parts used, functional components, pharmacological effects, mechanism of action, etc. Based on this assessment, Table 4: Characteristic metabolites of herbs used were established, clearly presenting the correspondence between each standardized plant entity and its functional components.

**TABLE 4:** Characteristic metabolite of herbs used.

Latin name (ChP 2020)	Functional ingredient	Chemical class	Pharmacopoeial content requirement (ChP 2020)	Pharmacological Effect(s)	Mechanism of action	References
*Morindae Officinalis Radix*	Physicion, Rubiadin	Anthraquinones	Not specified	Anti-osteoporotic	Promotes osteoblast differentiation; Inhibits RANKL/OPG pathway	Ru et al. (2014), Chen et al. (2023)
*Aconiti Lateralis Radix Praeparata*	Aconitine, Mesaconitine	Diterpenoid Alkaloids	Diester-diterpenoid alkaloids ≤0.015%	Cardiotonic, Analgesic	Activates Na^+^ channels; Enhances myocardial contractility	Ru et al., 2014; Gupta (2020)
*Bombyx Batryticatus*	Fibroin, Sericin	Structural Proteins	Total ash ≤7.0%	Antioxidant, Hypoglycemic	Scavenges ROS; Activates PI3K/Akt signaling pathway	Luo et al. (2024)
*Sinapis Semen*	Sinalbin	Glucosinolates	Not specified	Antibacterial, Anti-inflammatory	Hydrolyzes to generate phenethyl isothiocyanate; Inhibits NF-κB pathway	Chen et al. (2023)
*Pinelliae Rhizoma*	β-Sitosterol, Cavidine	Sterols, Alkaloids	Not specified	Antitumor (Lung cancer)	Induces G2/M phase arrest in cancer cells; Inhibits EGFR phosphorylation	Ru et al. (2014), Choi et al. (2022)
*Borneolum Syntheticum*	d-Borneol	Monoterpenoids	Borneol (C_10_H_18_O) ≥55.0%	Penetration enhancer, Anti-inflammatory	Enhances blood-brain barrier permeability; Inhibits COX-2 expression	Gupta (2020)
*Menthae Haplocalycis Herba*	Menthol, Menthone	Monoterpenoids	Volatile oil ≥0.8%	Antispasmodic, Antibacterial	Blocks calcium channels; Inhibits acetylcholine release	Ru et al. (2014)
*Atractylodis Macrocephalae Rhizoma*	Atractylenolide III	Sesquiterpene Lactones	Not specified	Anti-inflammatory, Immunomodulatory	Inhibits TNF-α/IL-6 secretion; Modulates Th1/Th2 balance	Choi et al. (2022)
*Chuanxiong Rhizoma*	Z-Ligustilide, Senkyunolide	Phthalides	Ferulic acid ≥0.10%	Antithrombotic, Neuroprotective	Inhibits TXA2 synthesis; Reduces cerebral ischemia-reperfusion injury	Choi et al. (2022)
*Fritillariae Cirrhosae Bulbus*	Peimine, Peimisine	Steroidal Alkaloids	Total alkaloids ≥0.080%	Antitussive, Expectorant	Inhibits airway sensory C-fibers; Reduces substance P release	Chen et al. (2023)
*Arisaema cum Bile*	Taurine, Cholic Acid	Bile Acids	Not specified	Antipyretic, Anticonvulsant	Modulates GABA_A receptors; Inhibits glutamate excitotoxicity	Gupta (2020)
*Pheretima*	Lumbrokinase	Protease	Not specified	Thrombolytic, Anticoagulant	Degrades fibrinogen; Inhibits platelet aggregation	Luo et al. (2024)
*Rehmanniae Radix*	Catalpol, Acteoside	Iridoid Glycosides	Catalpol ≥0.20%	Hypoglycemic, Anti-inflammatory	Activates GLUT4 translocation; Inhibits NF-κB nuclear translocation	Choi et al. (2022)
*Saposhnikoviae Radix*	Prim-O-Glucosylcimifugin	Chromones	Not specified	Antiallergic, Analgesic	Blocks histamine H1 receptors; Inhibits TRPV1 channel activation	Chen et al. (2023)
*Poria*	Pachymic Acid, Poriacosides	Triterpenoids	Not specified	Diuretic, Immunomodulatory	Inhibits renal tubular Na^+^-K^+^-ATPase; Enhances macrophage phagocytosis	Ru et al. (2014), Luo et al. (2024)
*Kansui Radix*	Kansuinin A	Diterpenoids	Not specified	Purgative, Antitumor	Activates enteric plexus NK1 receptors; Induces watery diarrhea	Chen et al. (2023)
*Glycyrrhizae Radix et Rhizoma*	Glycyrrhizin, Liquiritigenin	Triterpenoids, Flavonoids	Glycyrrhizic acid ≥2.0%	Antiulcer, Hepatoprotective	Inhibits 11β-HSD2; Reduces cortisol catabolism	Ru et al. (2014), Cao et al. (2022)
*Carthami Flos*	Hydroxysafflor yellow A	Chalcones	Not specified	Anti-myocardial ischemia, Anticoagulant	Inhibits PAF receptors; Reduces calcium influx	Choi et al. (2022)
*Astragali Radix*	Astragaloside IV, Calycosin	Saponins, Isoflavones	Astragaloside IV ≥ 0.040%	Immunostimulant, Antioxidant	Activates Nrf2 pathway; Enhances SOD activity	Ru et al. (2014), Choi et al. (2022)
*Platycodonis Radix*	Platycodin D	Triterpenoid Saponins	Not specified	Expectorant, Anti-inflammatory	Stimulates respiratory mucus secretion; Inhibits COX-2/PGE2 pathway	Chen et al. (2023)
*Citri Exocarpium Rubrum*	Hesperidin, Nobiletin	Flavonoids	Hesperidin ≥3.0%	Antioxidant, Digestive stimulant	Scavenges free radicals; Enhances gastrointestinal myenteric plexus activity	Ru et al. (2014), Choi et al. (2022)
*Liquidambaris Fructus*	Gallic Acid, Ellagic Acid	Phenolic Acids	Not specified	Antibacterial, Anti-inflammatory	Inhibits bacterial DNA gyrase; Blocks TLR4/MyD88 signaling	Luo et al. (2024)
*Ephedrae Herba*	Ephedrine, Pseudoephedrine	Alkaloids	Ephedrine ≥0.80%	Antiasthmatic, Diaphoretic	Agonizes β_2_-adrenergic receptors; Activates sweat gland Na^+^ channels	He et al. (2023)
*Ophiopogonis Radix*	Ophiopogonin D	Steroidal Saponins	Not specified	Anti-myocardial ischemia, Immunomodulatory	Enhances SOD activity; Inhibits TNF-α/IL-1β production	Choi et al. (2022)
*Achyranthis Bidentatae Radix*	β-Ecdysone, Inokosterone	Phytosterols	Not specified	Anti-arthritic, Osteogenic	Inhibits MMP-13 expression; Promotes osteoblast RUNX2 activation	Chen et al. (2023)
*Gentianae Macrophyllae Radix*	Gentiopicroside	Iridoids	Not specified	Anti-inflammatory, Hepatoprotective	Inhibits TGF-β1/Smad3 pathway; Attenuates hepatic fibrosis	Ru et al. (2014)
*Ginseng Radix et Rhizoma*	Ginsenoside Rg1, Rb1	Triterpenoid Saponins	Total saponins ≥0.30%	Anti-fatigue, Neuroprotective	Enhances mitochondrial complex I activity; Inhibits Aβ42 aggregation	Ru et al. (2014), Cao et al. (2022)
*Cistanches Herba*	Echinacoside	Phenylethanoid Glycosides	Echinacoside ≥0.30%	Aphrodisiac, Neuroprotective	Promotes NO/cGMP pathway; Inhibits tau protein hyperphosphorylation	Choi et al. (2022)
*Cinnamomi Cortex*	Cinnamaldehyde, Coumarin	Phenylpropanoids	Cinnamaldehyde ≥75.0%	Hypoglycemic, Antibacterial	Activates AMPK/GLUT4 pathway; Disrupts microbial cell membranes	Ru et al. (2014), He et al. (2023)
*Notoginseng Radix et Rhizoma*	Notoginsenoside R1	Triterpenoid Saponins	Ginsenoside Rg_1_ ≥ 0.20%	Hemostatic, Antithrombotic	Activates P2Y12 receptors; Promotes platelet aggregation	Chen et al. (2023)
*Taxilli Herba*	Quercetin, Avicularin	Flavonoids	Not specified	Anti-inflammatory, Antioxidant	Inhibits NF-κB nuclear translocation; Scavenges hydroxyl radicals	Choi et al. (2022)
*Corni Fructus*	Loganin, Cornuside	Iridoid Glycosides	Morroniside ≥0.20%	Hypoglycemic, Nephroprotective	Inhibits α-glucosidase; Reduces glomerular mesangial cell proliferation	Choi et al. (2022)
*Acori Tatarinowii Rhizoma*	β-Asarone	Phenylpropanoids	Not specified	Antiepileptic, Neuroprotective	Enhances GABAergic neurotransmission; Inhibits NMDA receptor overactivation	Ru et al. (2014)
*Dendrobii Caulis*	Dendrobine, Nobilonine	Alkaloids	Not specified	Immunomodulatory, Antitumor	Activates NK cell cytotoxicity; Induces cancer cell apoptosis	Luo et al. (2024)
*Moschus*	Muscone	Macrocyclic Ketones	Muscone ≥2.0%	Cardiotonic, Anti-inflammatory	Activates cardiomyocyte L-type calcium channels; Inhibits NLRP3 inflammasome	He et al. (2023)
*Styrax*	Styrax	Resin	Total vanillic acid ≥27.0%	Antibacterial, Expectorant	Disrupts bacterial biofilms; Stimulates respiratory ciliary motility	He et al. (2023)
*Persicae Semen*	Amygdalin	Cyanogenic Glycosides	Not specified	Antitumor, Anti-fibrotic	Inhibits TGF-β1/Smad pathway; Blocks EMT process	Choi et al. (2022)
*Schisandrae Chinensis Fructus*	Schisandrin B	Lignans	Schisandrin A ≥0.40%	Hepatoprotective, Antioxidant	Enhances GSH synthesis; Inhibits CYP450 enzyme activity	Ru et al. (2014), Choi et al. (2022)
*Asari Radix et Rhizoma*	Asarinin, Sesamin	Lignans	Volatile oil ≥2.0%	Analgesic, Anti-inflammatory	Inhibits COX-2/PGE2 pathway; Blocks TRPA1 nociceptive receptors	Choi et al. (2022)
*Scrophulariae Radix*	Harpagoside	Iridoid Glycosides	Harpagoside ≥0.050%	Anti-inflammatory, Immunosuppressive	Inhibits NF-κB activation; Reduces Th17 cell differentiation	Cao et al. (2022)
*Polygalae Radix*	Tenuifolin	Saponins	Not specified	Antidepressant, Neuroprotective	Upregulates BDNF/TrkB pathway; Inhibits MAO-A activity	Ru et al. (2014), Choi et al. (2022)
*Aurantii Fructus*	Synephrine, Naringin	Alkaloids, Flavonoids	Synephrine ≥0.30%	Digestive stimulant, Pressor	Agonizes α_1_-adrenergic receptors; Enhances gastrointestinal motility	Choi et al. (2022)
*Bambusae Caulis in Taenias*	Bambooside A	Flavonoid Glycosides	Not specified	Antioxidant, Antitussive	Scavenges superoxide anions; Inhibits airway hyperresponsiveness	Luo et al. (2024)
*Angelicae Sinensis Radix*	Z-Ligustilide, Ferulic Acid	Phthalides, Phenolic Acids	Ferulic acid ≥0.050%	Hematopoietic, Anticoagulant	Promotes EPO secretion; Inhibits platelet TXA2 synthesis	Choi et al. (2022)
*Paeoniae Radix Rubra*	Paeoniflorin	Monoterpene Glycosides	Paeoniflorin ≥1.8%	Antispasmodic, Anti-inflammatory	Inhibits PGE2 synthesis; Blocks calcium influx	Ru et al. (2014), Choi et al. (2022)
*Bovis Calculus*	Cholic Acid, Tauroursodeoxycholic Acid	Bile Acids	Cholic acid ≥13.0%	Antipyretic, Anticonvulsant	Activates FXR receptors; Inhibits TLR4/NF-κB pathway	He et al. (2023)
*Bufonis Venenum*	Bufalin, Cinobufagin	Steroidal Lactones	Cinobufagin ≥6.0%	Antitumor, Cardiotonic	Inhibits Na^+^/K^+^-ATPase; Activates JNK apoptotic pathway	Chen et al. (2023)

Collectively, these two tables form the core output of the scientific definition, component standardization, and taxonomic validation of SETCM therapy interventions in this study, providing a reliable, consistent foundation for subsequent analyses (such as efficacy evaluation and mechanistic exploration).

### 3.3 Quality assessment (risk of bias) of included studies

**FIGURE 2:**
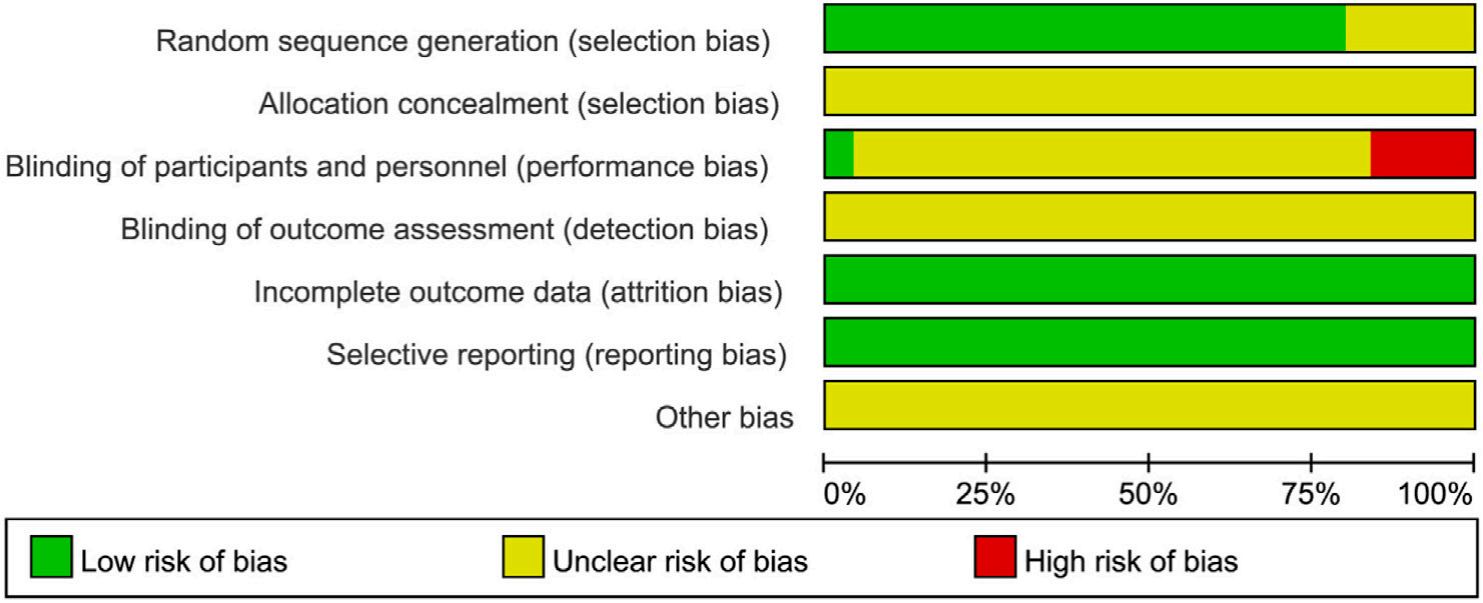
Results of the risk of bias assessment of the included literature.

**FIGURE 3:**
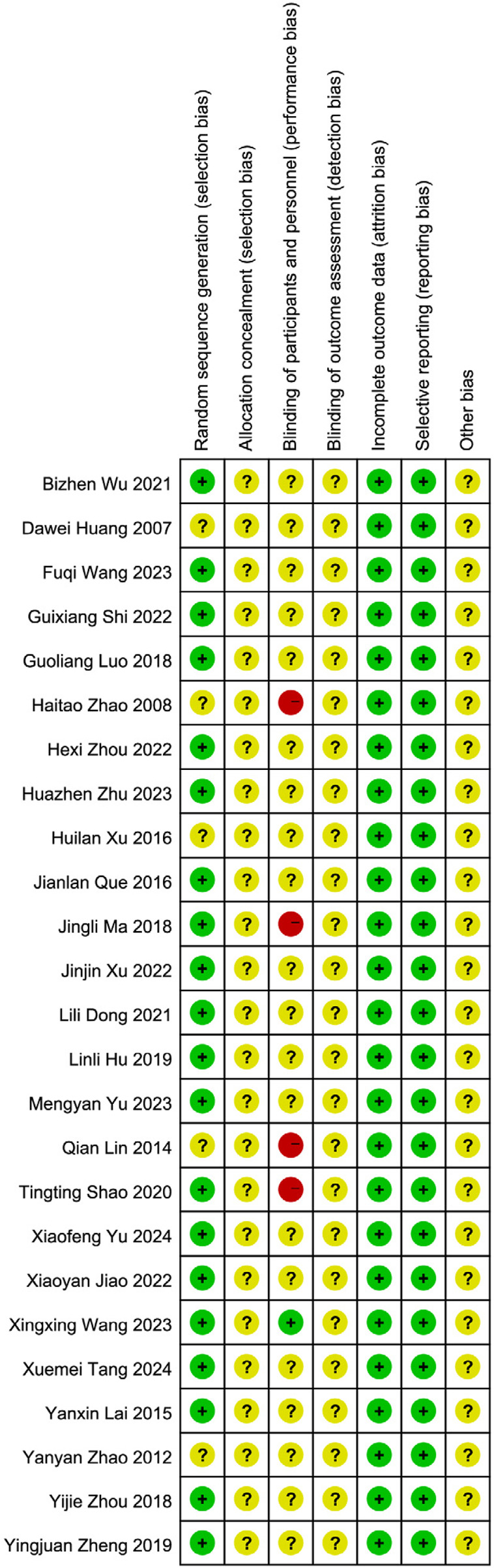
Summary of the risk of bias of the included literature.

All 25 included studies (Heinrich et al., 2018; Guyatt et al., 2011; Tang and Liu, 2024; Yu and Wu, 2024; Wang and Sun, 2023; Zhu et al., 2023; Yu and Qiu, 2023; Wang FQ. et al., 2023; Xu, 2022; Shi et al., 2022; Zhou et al., 2022; Jiao, 2022; Wu et al., 2021; Dong et al., 2021; Shao and Chen, 2020; Zheng et al., 2019; Zhou et al., 2018; Luo et al., 2018; Ma et al., 2021; Xu and Zhao, 2016; Que and Bian, 2016; Lai, 2015; Lin et al., 2014; Zhao et al., 2012; Zhao et al., 2008) were published randomized controlled trials (RCTs) involving patients with PSD. The risk of bias across seven domains was rigorously assessed using the Cochrane RoB 1.0 tool, with complete study-level judgments detailed in Table 5. The risk of bias assessment was as follows: Randomization: Five studies (Ma et al., 2021; Lai, 2015; Lin et al., 2014; Zhao et al., 2012; Zhao et al., 2008) did not mention a specific randomization scheme (Unclear risk), while the remainder described randomization methods (Low risk). Allocation concealment: No studies provided sufficient information (Unclear risk). Blinding: Four studies (Wu et al., 2021; Luo et al., 2018; Lai, 2015; Zhao et al., 2012) did not implement blinding (High risk); One study (Tang and Liu, 2024) implemented blinding (Low risk); the remainder were Unclear risk. Attrition bias: All studies reported no dropouts (Low risk). Reporting bias: No selective reporting was detected (Low risk). Other bias: Insufficient data (Unclear risk). See Figures 2, 3. Although the 25 included RCTs collectively showed positive efficacy signals, significant methodological limitations existed: Allocation concealment was inadequately described in all studies (100% “Unclear risk”); Blinding of participants and personnel was High risk in 16% of studies (4/25); Blinding of outcome assessment was Unclear risk in 92% of studies (23/25). These deficiencies, particularly the lack of allocation concealment and blinding, may introduce selection bias and performance/detection bias, potentially inflating the observed treatment effects.

**TABLE 5:** Bias risk assessment table.

Author(s)	Random sequence generation	Allocation concealment	Blinding of participants and personnel	Blinding of outcome assessment	Incomplete outcome data	Selective reporting	Other bias
Tang and Liu (2024) Heinrich et al. (2018)	Low risk	Unclear risk	Unclear risk	Unclear risk	Low risk	Low risk	Unclear risk
Yu and Wu (2024)	Low risk	Unclear risk	Unclear risk	Unclear risk	Low risk	Low risk	Unclear risk
Wang and Sun (2023)	Low risk	Unclear risk	Unclear risk	Unclear risk	Low risk	Low risk	Unclear risk
Zhu et al. (2023)	Low risk	Unclear risk	Unclear risk	Unclear risk	Low risk	Low risk	Unclear risk
Yu and Qiu (2023)	Low risk	Unclear risk	Unclear risk	Unclear risk	Low risk	Low risk	Unclear risk
Wang et al. (2023b)	Low risk	Unclear risk	Unclear risk	Unclear risk	Low risk	Low risk	Unclear risk
Xu (2022)	Low risk	Unclear risk	Unclear risk	Unclear risk	Low risk	Low risk	Unclear risk
Shi et al. (2022)	Low risk	Unclear risk	Unclear risk	Unclear risk	Low risk	Low risk	Unclear risk
Zhou et al. (2022)	Low risk	Unclear risk	Unclear risk	Unclear risk	Low risk	Low risk	Unclear risk
Jiao (2022)	Low risk	Unclear risk	Unclear risk	Unclear risk	Low risk	Low risk	Unclear risk
Wu et al. (2021)	Low risk	Unclear risk	Unclear risk	Unclear risk	Low risk	Low risk	Unclear risk
Dong et al. (2021)	Low risk	Unclear risk	Unclear risk	Unclear risk	Low risk	Low risk	Unclear risk
Shao and Chen (2020)	Low risk	Unclear risk	High risk	Unclear risk	Low risk	Low risk	Unclear risk
Zheng et al. (2019)	Low risk	Unclear risk	Unclear risk	Unclear risk	Low risk	Low risk	Unclear risk
Zhou et al. (2018) Shao and Chen (2020)	Low risk	Unclear risk	Unclear risk	Unclear risk	Low risk	Low risk	Unclear risk
Luo et al. (2018)	Low risk	Unclear risk	Unclear risk	Unclear risk	Low risk	Low risk	Unclear risk
Ma et al. (2021)	Low risk	Unclear risk	Unclear risk	Unclear risk	Low risk	Low risk	Unclear risk
Xu and Zhao (2016)	Low risk	Unclear risk	High risk	Unclear risk	Low risk	Low risk	Unclear risk
Que and Bian (2016)	Unclear risk	Unclear risk	Unclear risk	Unclear risk	Low risk	Low risk	Unclear risk
Lai (2015) Xu and Zhao (2016)	Low risk	Unclear risk	Unclear risk	Unclear risk	Low risk	Low risk	Unclear risk
Lin et al. (2014)	Low risk	Unclear risk	Unclear risk	Unclear risk	Low risk	Low risk	Unclear risk
Zhao et al. (2012)	Unclear risk	Unclear risk	High risk	Unclear risk	Low risk	Low risk	Unclear risk
Zhao et al. (2008)	Unclear risk	Unclear risk	Unclear risk	Unclear risk	Low risk	Low risk	Unclear risk
Huang (2007)	Unclear risk	Unclear risk	High risk	Unclear risk	Low risk	Low risk	Unclear risk
Yuan et al. (2024)	Unclear risk	Unclear risk	Unclear risk	Unclear risk	Low risk	Low risk	Unclear risk

### 3.4 Safety monitoring and adverse event reporting

This study systematically extracted and re-evaluated all reported adverse events (AEs) and serious adverse events (SAEs) from the included primary studies (n = 25 RCTs). Safety data were categorized and graded according to the US National Cancer Institute Common Terminology Criteria for Adverse Events version 5.0 (CTCAE v5.0). The specific process was as follows.

#### 3.4.1 AE/SAE extraction and verification

Two researchers independently extracted explicitly reported AEs/SAEs from each study, including event type, number of occurrences, severity, causality relationship to the study intervention, and management measures. For vaguely described events (e.g., “local discomfort”), the original authors were contacted for clarification, and if details were unobtainable, the event was marked as “Unspecified.”

#### 3.4.2 CTCAE v5.0 standardized classification

All AEs were classified by organ system and graded for severity into five levels:

Grade 1 (Mild): Asymptomatic or mild symptoms; intervention not indicated. Grade 2 (Moderate): Minimal, local, or noninvasive intervention indicated. Grade 3 (Severe): Hospitalization or prolongation of hospitalization indicated; disabling; limiting self-care activities of daily living (ADL). Grade 4 (Life-threatening): Urgent intervention indicated. Grade 5 (Death): Death related to AE.

#### 3.4.3 Safety analysis results

Only three studies (12% of included studies) reported AEs. All reported events were local skin reactions, with a cumulative occurrence of 24 cases (Total sample size N = 2,159), yielding an overall incidence rate of 2.1%. The detailed classification is as follows (Table 6).

**TABLE 6:** Adverse events associated with external herbal treatment method (classified according to CTCAE v5.0).

Organ system	Type of AE	CTCAE terminology	Severity	Number of occurrences	Source of study	Did it lead to the interruption of the intervention
Skin and subcutaneous tissues	Localized erythema	Rash maculo-papular	Grade 1	12	Yu Mengyan, 2023	No
Skin and subcutaneous tissues	Pruritus	Pruritus	Grade 1	8	Shao Tingting, 2020	No
Skin and subcutaneous tissues	Contact dermatitis	Dermatitis contact	Grade 2	4	Zhu Huazhen, 2023	Yes (2 cases with patch suspension)

Key Findings: The only reported AE type: Skin and subcutaneous tissue disorders (100%). Severity distribution:

Grade 1 (83.3%, n = 20): Transient erythema/pruritus; resolved spontaneously without intervention.

Grade 2 (16.7%, n = 4): Contact dermatitis; required temporary intervention suspension and topical corticosteroid application.

No SAEs reported: No Grade 3–5 events or systemic reactions (e.g., allergy, hepatic/renal dysfunction) occurred.

#### 3.4.4 Evidence limitations

Incomplete reporting: 88% of studies (22/25) failed to describe AE monitoring procedures or explicitly state “no adverse reactions observed.”

Unclear causality: No studies provided laboratory-confirmed causal links (e.g., patch testing) between AEs and specific herbal constituents/excipients.

Lack of long-term safety data: All studies omitted follow-up for delayed reactions (>4 weeks post-intervention). Most (88%) studies lacked prospective safety monitoring protocols, precluding definitive conclusions about long-term tolerability.

### 3.5 Meta-analysis results

#### 3.5.1 Meta-analysis of overall response rate

**FIGURE 4:**
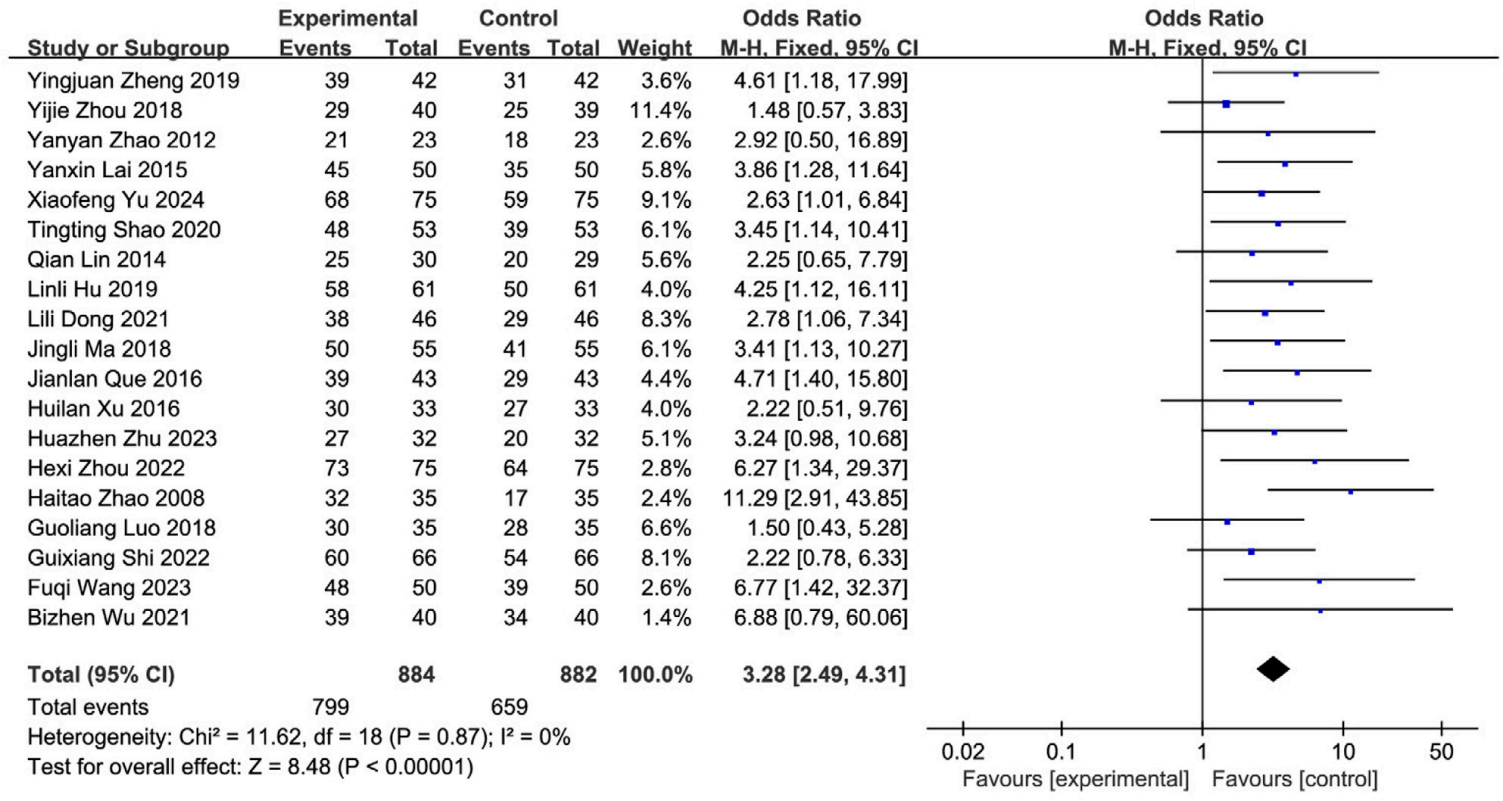
Meta-analysis of the total clinical effectiveness rate of the 19 studies of SETCM therapies for PSD included.

**FIGURE 5:**
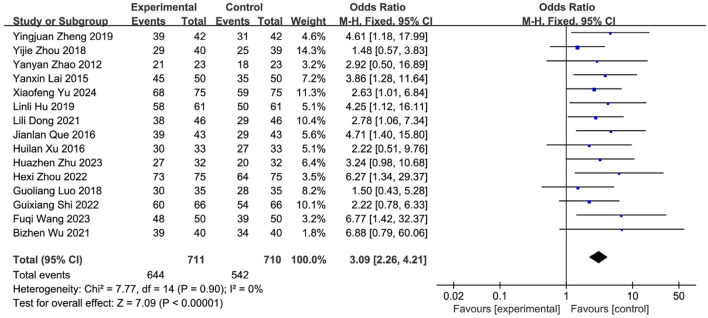
Sensitivity analysis of the total clinical effectiveness rate of the 15 studies of SETCM therapies for PSD included.

Nineteen studies (Yu and Wu, 2024; Zhu et al., 2023; Wang FQ. et al., 2023; Shi et al., 2022; Zhou et al., 2022; Wu et al., 2021; Dong et al., 2021; Shao and Chen, 2020; Zheng et al., 2019; Zhou et al., 2018; Luo et al., 2018; Ma et al., 2021; Xu and Zhao, 2016; Que and Bian, 2016; Lai, 2015; Lin et al., 2014; Zhao et al., 2012; Zhao et al., 2008; Huang, 2007) reported the post-treatment clinical overall response rate, involving 1,766 patients (SETCM therapies group: n = 884, effective cases = 799; Control group: n = 882, effective cases = 659). Heterogeneity testing (I^2^ = 0%, P = 0.87) indicated homogeneity among the selected studies, warranting the use of a fixed-effects model (FEM) for meta-analysis. The analysis revealed a statistically significant difference in the overall response rate between the SETCM therapies group and the control group [OR = 3.28, 95% CI: (2.49, 4.31), P < 0.00001]. This indicates that the application of SETCM therapies significantly improved the clinical overall response rate compared to the control group (Figure 4). Sensitivity analysis, excluding three studies (Shao and Chen, 2020; Xu and Zhao, 2016; Huang, 2007) rated as high risk for performance bias (blinding of participants), showed that the direction and statistical significance of the pooled effect estimate remained materially unchanged [OR = 3.09, 95% CI: (2.26, 4.21), P < 0.00001], demonstrating the robustness of the meta-analysis results (Figure 5).

#### 3.5.2 Meta-analysis of cure rate

**FIGURE 6:**
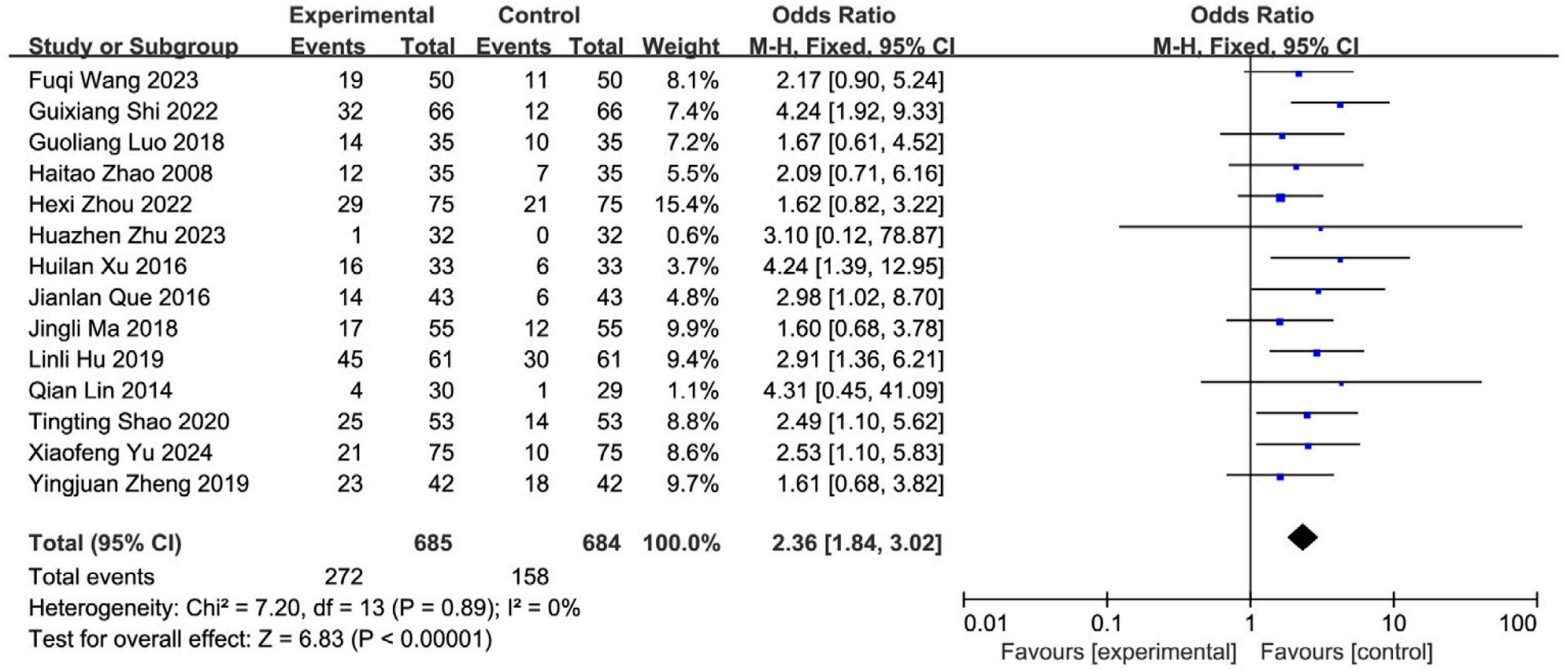
Meta-analysis of the cure rate of the 14 included studies of SETCM therapies for PSD.

Fourteen studies (Yu and Wu, 2024; Zhu et al., 2023; Wang FQ. et al., 2023; Shi et al., 2022; Zhou et al., 2022; Shao and Chen, 2020; Zheng et al., 2019; Zhou et al., 2018; Ma et al., 2021; Xu and Zhao, 2016; Que and Bian, 2016; Lai, 2015; Zhao et al., 2012; Huang, 2007) reporting the post-treatment clinical cure rate were included, involving 1,369 patients (SETCM therapies group: n = 685, cured cases = 272; Control group: n = 684, cured cases = 158). Heterogeneity testing (I^2^ = 0%, P = 0.89) indicated homogeneity among the selected studies, warranting the use of an FEM for meta-analysis. The analysis revealed a statistically significant difference in the cure rate between the SETCM therapies group and the control group [OR = 2.36, 95% CI: (1.84, 3.02), P < 0.00001]. This indicates that the application of SETCM therapies significantly improved the clinical cure rate compared to the control group (Figure 6).

#### 3.5.3 Meta-analysis of water swallowing test (WST) scores

**FIGURE 7:**
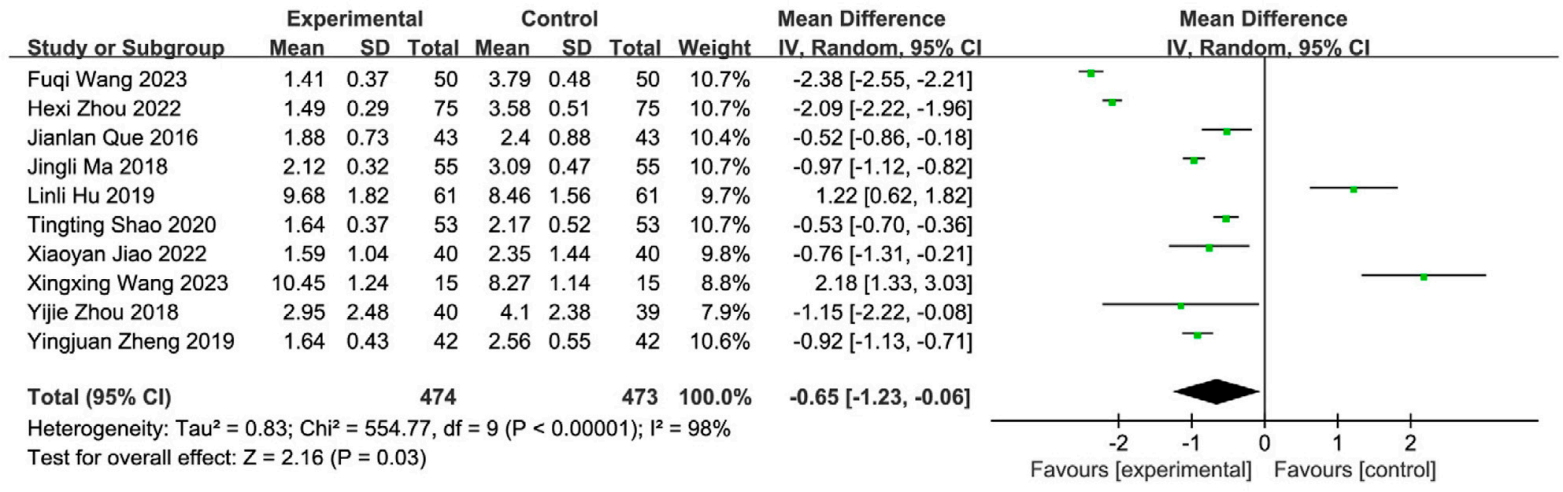
Meta-analysis of WST scores of 10 included studies of SETCM therapies for PSD.

Ten studies (Wang and Sun, 2023; Wang FQ. et al., 2023; Jiao, 2022; Shao and Chen, 2020; Zheng et al., 2019; Zhou et al., 2018; Luo et al., 2018; Xu and Zhao, 2016; Que and Bian, 2016; Lai, 2015) reported post-treatment WST scores, involving 947 patients (SETCM therapies group: n = 474; Control group: n = 473). Overall heterogeneity testing indicated substantial heterogeneity (I^2^ = 98%, P < 0.00001). Sequential removal of individual studies failed to significantly reduce heterogeneity, suggesting intervention modality as a primary source. A random-effects model (REM) was employed for meta-analysis. Results demonstrated that SETCM therapies significantly reduced WST scores compared to controls [MD = −0.65, 95%CI: (−1.23, −0.06), P = 0.03] (Figure 7). High heterogeneity likely originated from variations in intervention type, duration, assessment timing, and combination therapies.

**FIGURE 8:**
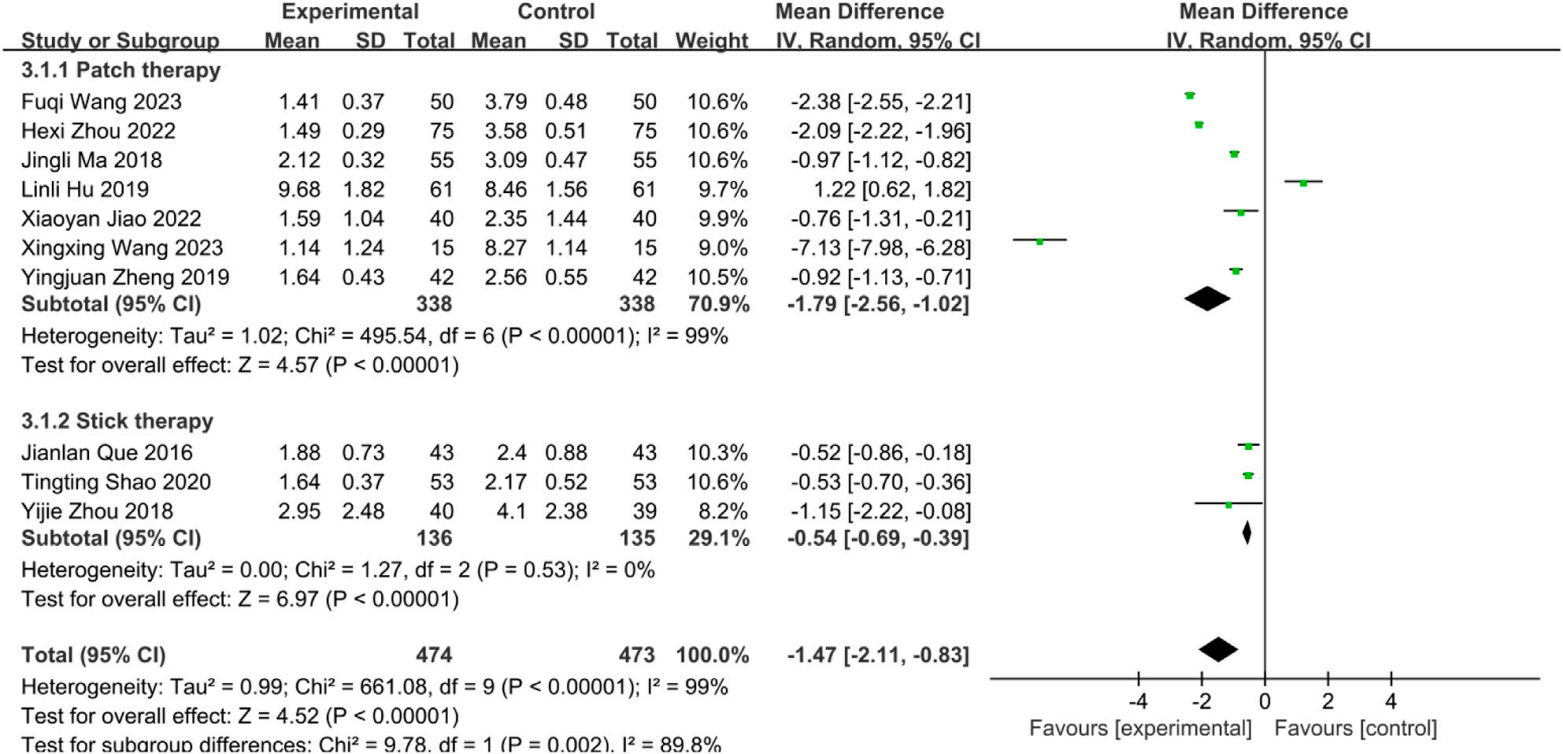
Subgroup analysis of WST scores of 10 included studies of SETCM therapies for PSD.

Subgroup analysis by intervention type revealted significant residual heterogeneity (I^2^ = 89.8%, P = 0.002), necessitating REM. Results showed: Herbal patching: Significantly reduced WST scores [MD = −1.79, 95%CI: (−2.56, −1.02), P < 0.00001]. Medicated stick stimulation: Significantly reduced WST scores [MD = −0.54, 95%CI: (−0.69, −0.39), P < 0.00001] (Figure 8). While all subgroups demonstrated efficacy, unresolved heterogeneity suggests contributions from technical factors (operator skill, application site, session duration, intervention timing).

#### 3.5.4 Meta-analysis of videofluoroscopic swallow study (VFSS) scores

**FIGURE 9:**
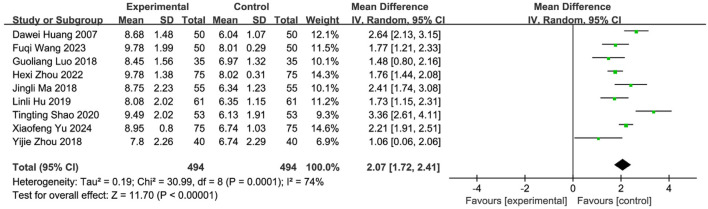
Meta-analysis of VFSS scores of nine included studies examining SETCM therapies for PSD.

**FIGURE 10:**
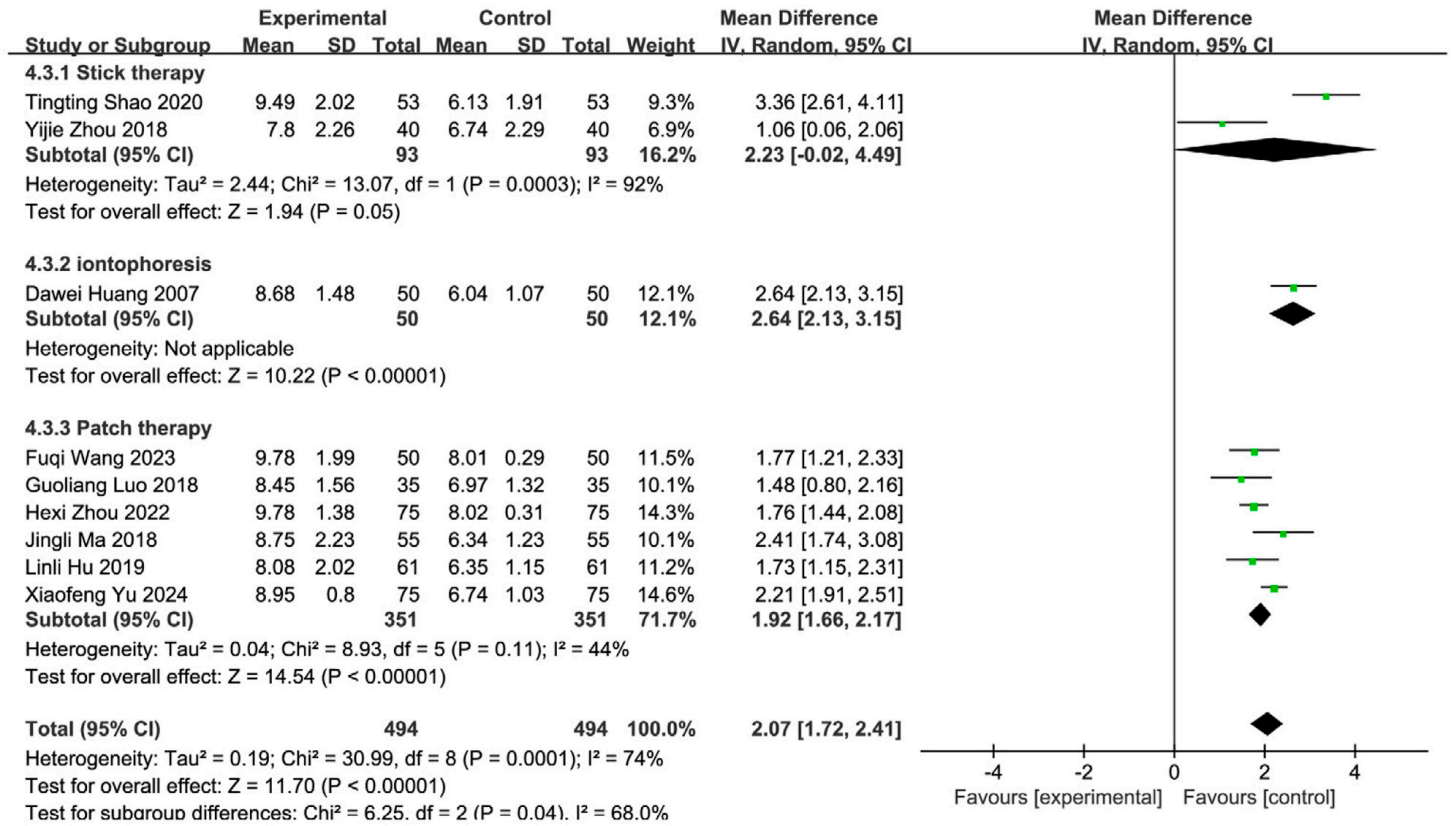
Subgroup analysis of VFSS scores of nine included studies of SETCM therapies for PSD.

Nine studies (Yu and Wu, 2024; Wang FQ. et al., 2023; Zhou et al., 2022; Shao and Chen, 2020; Zhou et al., 2018; Luo et al., 2018; Ma et al., 2021; Xu and Zhao, 2016; Yuan et al., 2024) involving 988 PSD patients (SETCM therapies: n = 494; Control: n = 494) evaluated swallowing function using VFSS scores. Random-effects meta-analysis indicated SETCM therapies significantly improved VFSS scores versus controls [MD = 2.07, 95%CI (1.72, 2.41), Z = 11.70, P < 0.00001], with substantial heterogeneity (I^2^ = 74%, P = 0.0001; Figure 9). Subgroup analysis by intervention type (Figure 10).

**FIGURE 11:**
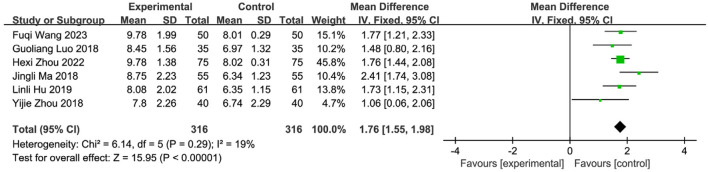
SETCM therapy sensitivity analysis of VFSS scores of six included studies of the treatment of PSD.

Medicated stick (two studies): Moderate improvement [MD = 2.23, 95%CI (−0.02, 4.49)], high heterogeneity (I^2^ = 92%, P = 0.0003). Iontophoresis (one study): Significant improvement [MD = 2.64, 95%CI (2.13, 3.15)]. Herbal patching (six studies): Consistent improvement [MD = 1.92, 95%CI (1.66, 2.17)], moderate heterogeneity (I^2^ = 44%, P = 0.11). Subgroup differences were significant (P = 0.04, I^2^ = 68.0%), confirming that intervention type partially explained heterogeneity. Sensitivity analysis identified three influential studies (Yu and Wu, 2024; Shao and Chen, 2020; Yuan et al., 2024) (potential confounders: patient age, operator technique, treatment duration/timing). Their exclusion reduced heterogeneity (I^2^ = 19%, P = 0.29). Fixed-effects analysis affirmed robust efficacy [MD = 1.75, 95%CI (1.55, 1.98), Z = 15.95, P < 0.00001; Figure 11]. SETCM therapies significantly enhanced VFSS scores and accelerated swallowing recovery.

#### 3.5.5 Meta-analysis of SWAL-QOL scores

**FIGURE 12:**
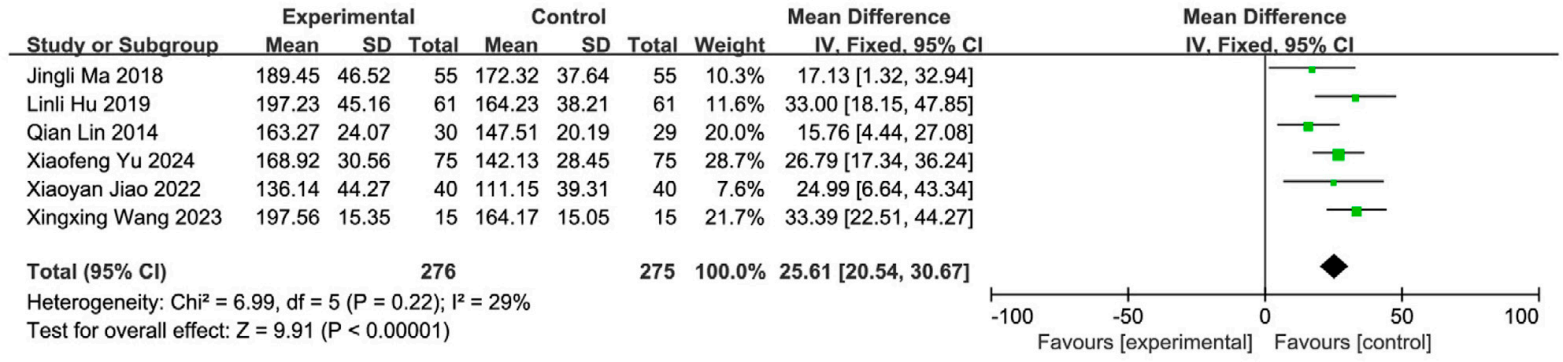
Meta-analysis of SWAL-QOL scores of six included studies of the treatment of PSD using SETCM therapies.

Six studies (Yu and Wu, 2024; Wang and Sun, 2023; Jiao, 2022; Zhou et al., 2018; Xu and Zhao, 2016; Zhao et al., 2012) reported SWAL-QOL scores, involving 551 patients (SETCM therapies: n = 275; Control: n = 276). Heterogeneity testing (I^2^ = 29%, P = 0.22) indicated homogeneity, supporting an FEM. Meta-analysis demonstrated a statistically significant improvement in the SETCM therapies group [MD = 25.61, 95%CI (20.54, 30.67), Z = 9.91, P < 0.00001], indicating SETCM therapies significantly reduced SWAL-QOL scores (i.e., improved quality of life) (Figure 12).

#### 3.5.6 Meta-analysis of post-intervention aspiration

**FIGURE 13:**
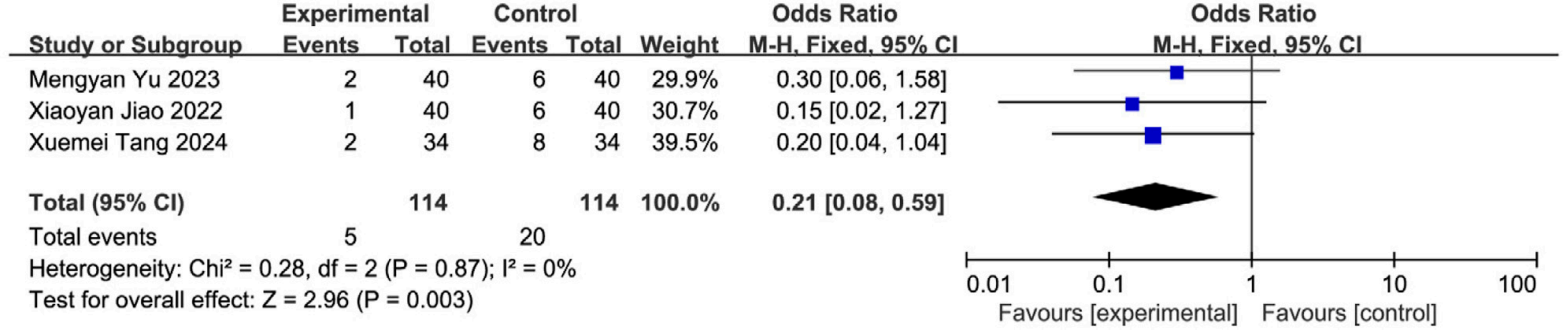
Meta-analysis of misabsorption rates of three included studies on the treatment of PSD by SETCM therapies.

Three studies (Tang and Liu, 2024; Yu and Qiu, 2023; Jiao, 2022) reported aspiration events, involving 228 patients (SETCM therapies: n = 114, aspiration = 6; Control: n = 114, aspiration = 20). Homogeneity was confirmed (I^2^ = 0%, P = 0.87), warranting an FEM. SETCM therapies significantly reduced aspiration risk [OR = 0.21, 95%CI (0.08, 0.59), Z = 2.96, P = 0.003] (Figure 13).

#### 3.5.7 Supplementary meta-analyses: Activities of daily living

##### 3.5.7.1 MBI score meta-analysis

**FIGURE 14:**
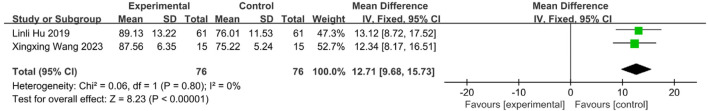
Meta-analysis of MBI scores of two included studies on the treatment of PSD by SETCM therapies.

Two studies (Wang and Sun, 2023; Zhou et al., 2018) reported MBI scores (n = 152; SETCM therapies:76, Control:76). Homogeneity (I^2^ = 0%, P = 0.72) supported FEM. SETCM therapies significantly improved MBI scores [MD = 12.71, 95%CI (9.68, 15.73), Z = 8.23, P < 0.00001], indicating enhanced daily living independence (Figure 14).

##### 3.5.7.2 ADL score meta-analysis

**FIGURE 15:**
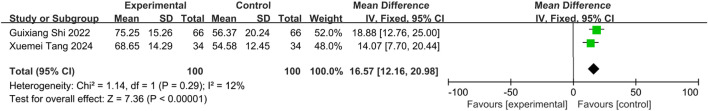
Meta-analysis of two included studies reporting ADL scores for treatment of PSD with SETCM therapies.

Two studies (Tang and Liu, 2024; Shi et al., 2022) reported ADL scores (n = 200; SETCM therapies:100, Control:100). Homogeneity (I^2^ = 12%, P = 0.29) supported FEM. SETCM therapies significantly improved ADL scores [MD = 16.57, 95%CI (12.16, 20.98), Z = 7.36, P < 0.00001], confirming better self-care capacity (Figure 15).

#### 3.5.8 Meta-analysis of serum albumin (ALB) levels

**FIGURE 16:**
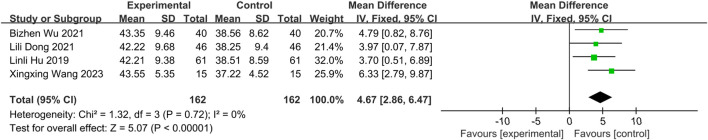
Meta-analysis of four included studies of ALB levels for SETCM therapy treatment of PSD.

Four studies (Wang and Sun, 2023; Wu et al., 2021; Dong et al., 2021; Zhou et al., 2018) involving 324 patients (intervention: n = 162; control: n = 162) reported serum albumin (ALB) levels. Homogeneity was confirmed (*I* ^2^ = 0%, *P* = 0.72), and an FEM was applied. Results demonstrated significantly higher ALB levels in the intervention group than in the controls [MD = 4.67, 95% CI (2.86, 6.47), *Z* = 5.07, *P* < 0.00001], indicating that SETCM therapies increase serum albumin concentrations in patients with PSD (Figure 16).

#### 3.5.9 Meta-analysis of prealbumin (PA) levels

**FIGURE 17:**
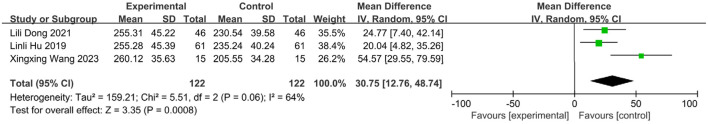
Meta-analysis of three included studies on PA levels during SETCM therapy treatment of PSD.

**FIGURE 18:**
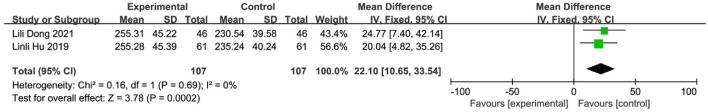
Sensitivity analysis of two included studies on PA levels during SETCM therapy treatment of PSD.

Three studies (Wang and Sun, 2023; Dong et al., 2021; Zhou et al., 2018) involving 244 patients (intervention: n = 122; control: n = 122) reported prealbumin (PA) levels. Significant heterogeneity was detected (*I* ^2^ = 64%, *P* = 0.06), necessitating a random-effects model (REM) (Figure 17). Sensitivity analysis identified Wang Xingxing’s study (Wang and Sun, 2023) as the source of heterogeneity; its exclusion resulted in homogeneity (*I* ^2^ = 0%, *P* = 0.69). The observed heterogeneity likely originated from the relatively small sample size in Wang and Sun (2023), which may compromise statistical power. An FEM was applied to the remaining two homogeneous studies. Results showed a statistically significant increase in PA levels for the intervention group [MD = 22.10, 95% CI (10.65, 33.54), *Z* = 3.78, *P* = 0.0002], demonstrating that SETCM therapies elevate prealbumin levels in PSD patients (Figure 18).

#### 3.5.10 Meta-analysis of NRS2002 scores

**FIGURE 19:**
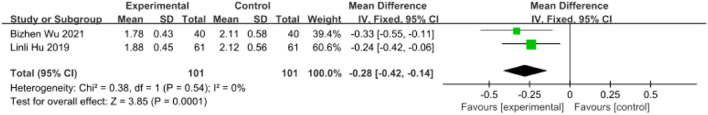
Meta-analysis of two included studies of SETCM therapy treatment of PSD, Meta-analysis of literature NRS2002 scores.

Two studies (Wu et al., 2021; Zhou et al., 2018) involving 202 patients (intervention: n = 101; control: n = 101) reported Nutritional Risk Screening 2002 (NRS 2002) scores. Homogeneity was confirmed (*I* ^2^ = 0%, *P* = 0.54), supporting an FEM. Results revealed a statistically significant reduction in nutritional risk scores for the intervention group [MD = −0.28, 95% CI (−0.42, −0.14), *Z* = 3.85, *P* = 0.0001], suggesting that SETCM therapies mitigate malnutrition risk in PSD patients (Figure 19).

#### 3.5.11 Meta-analysis of GUSS scores

**FIGURE 20:**
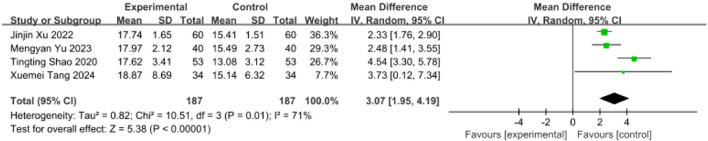
Meta-analysis of GUSS scores from four included studies of SETCM therapies for PSD.

**FIGURE 21:**
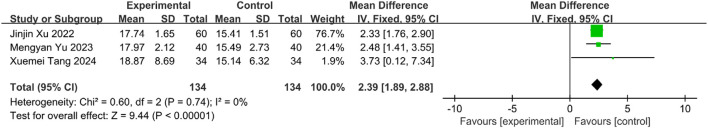
Sensitivity analysis of GUSS scores from three included studies of SETCM therapies for PSD.

Four studies (Tang and Liu, 2024; Yu and Qiu, 2023; Xu, 2022; Shao and Chen, 2020) involving 374 patients (intervention: n = 187; control: n = 187) reported GUSS scores. Heterogeneity testing indicated substantial variability among studies (*I* ^2^ = 71%, *P* = 0.01), warranting a random-effects model (REM) for meta-analysis (Figure 20). Sensitivity analysis via sequential exclusion revealed that removal of Shao Tingting’s study (Shao and Chen, 2020) significantly reduced heterogeneity (*I* ^2^ = 0%, *P* = 0.74). This observed heterogeneity likely stemmed from divergent intervention protocols in Shao and Chen (2020), which increased the dispersion of effect estimates. The remaining three homogeneous studies were analyzed using an FEM. Results demonstrated a statistically significant improvement in GUSS scores for the intervention group [MD = 2.39, 95% CI (1.89, 2.88), *Z* = 9.44, *P* < 0.00001], indicating that SETCM therapies significantly enhance swallowing function in PSD patients (Figure 21).

#### 3.5.12 Meta-analysis of enteral nutrition tube duration

**FIGURE 22:**
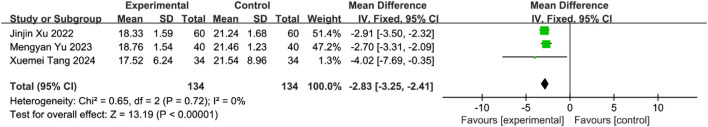
Meta-analysis of gastric tube retention duration of three included studies of SETCM therapies for PSD.

Three studies (Tang and Liu, 2024; Yu and Qiu, 2023; Xu, 2022) involving 268 patients (intervention: n = 134; control: n = 134) reported enteral nutrition tube duration. Heterogeneity testing confirmed homogeneity (*I* ^2^ = 0%, *P* = 0.72), supporting an FEM. Results showed a statistically significant reduction in tube duration for the intervention group [MD = −2.83, 95% CI (−3.25, −2.41), *Z* = 13.19, *P* < 0.00001], indicating that SETCM therapies shorten enteral nutrition dependency in PSD patients (Figure 22).

#### 3.5.13 Meta-analysis of tube removal success rate

**FIGURE 23:**
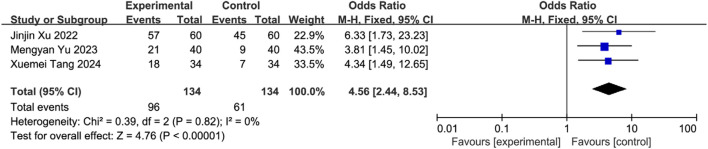
Gastric tube removal success rates reported in three included studies on SETCM therapies for PSD. Meta-analysis of gastric tube removal success rates in the literature.

Three studies (Tang and Liu, 2024; Yu and Qiu, 2023; Xu, 2022) involving 268 patients (intervention: n = 134; control: n = 134) reported tube removal success rates. Homogeneity was confirmed (*I* ^2^ = 0%, *P* = 0.82), and an FEM was applied. Results demonstrated a statistically significant increase in success rates for the intervention group [OR = 4.56, 95% CI (2.44, 8.53), *Z* = 4.76, *P* < 0.00001], indicating that SETCM therapies improve enteral nutrition tube removal outcomes in PSD patients (Figure 23).

#### 3.5.14 Meta-analysis of FDS scores

**FIGURE 24:**
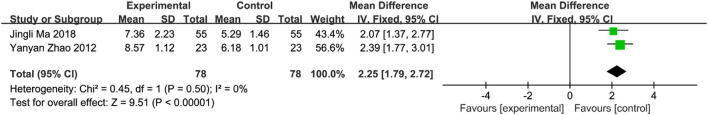
Meta-analysis of FDS dysphagia efficacy score reported by two included studies of SETCM therapies for PSD hemispheres.

Two studies (Xu and Zhao, 2016; Zhao et al., 2008) involving 156 patients (intervention: n = 78; control: n = 78) reported functional dysphagia scale (FDS) scores. Heterogeneity testing confirmed homogeneity (*I* ^2^ = 0%, *P* = 0.50), supporting an FEM. Results demonstrated statistically significant between-group differences [MD = 2.25, 95% CI (1.79, 2.72), *Z* = 9.51, *P* < 0.00001], indicating that SETCM therapies improve swallowing function in PSD patients (Figure 24).

#### 3.5.15 Stratified efficacy analysis by stroke location and intervention modality

**FIGURE 25:**
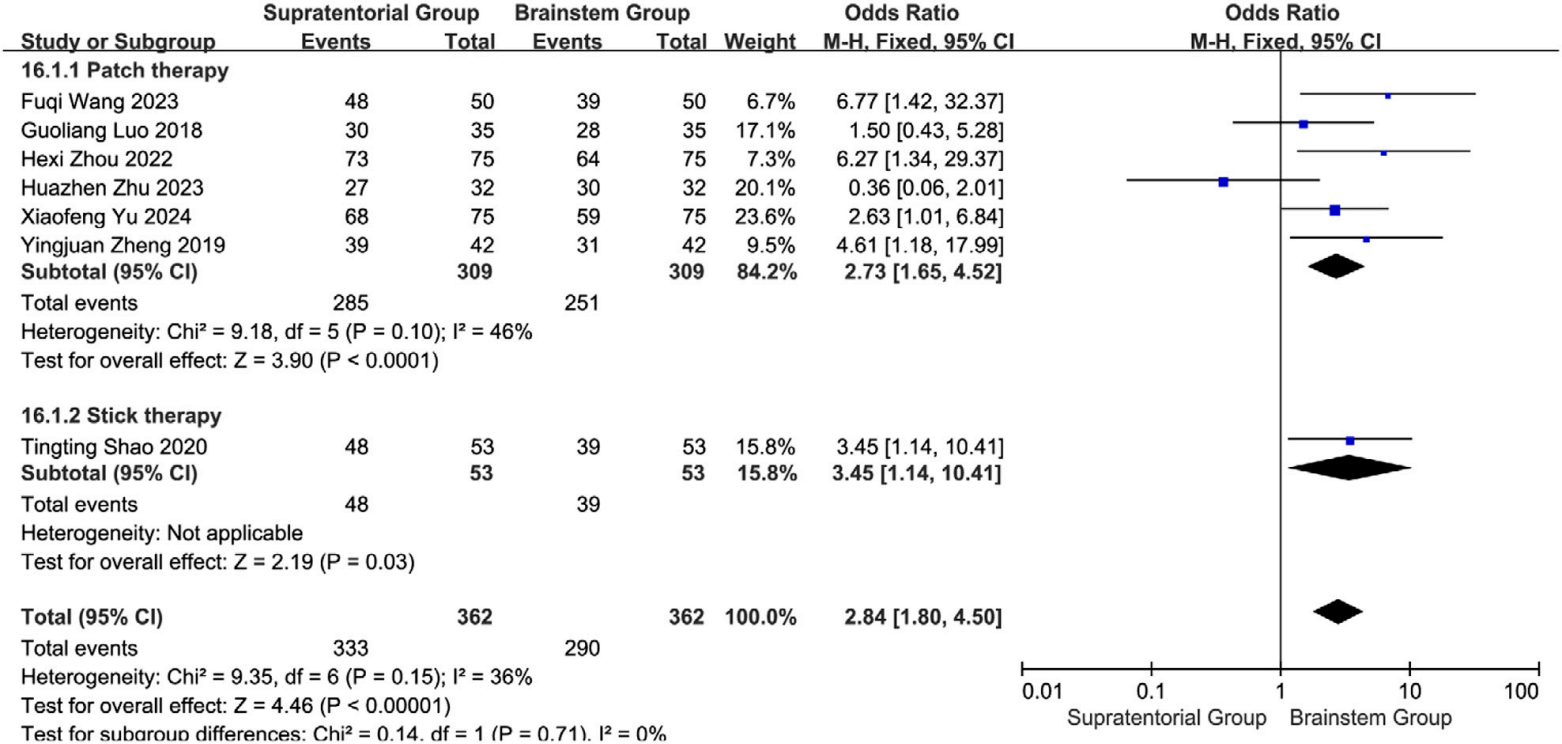
Subgroup analysis of the efficacy of different interventions in patients with stroke foci located in the cerebral hemispheres.

Considering potential effect variations across interventions and lesion locations, efficacy outcomes were stratified by stroke site (supratentorial vs. brainstem) and intervention type. Supratentorial subgroup analysis: Acupoint application showed significant efficacy: Pooled OR = 2.73 (95% CI: 1.65–4.52, *P* < 0.0001) with low heterogeneity (*I* ^2^ = 46%, *P* = 0.10). Wang Fuqi (2023): OR = 6.77 [1.42, 32.37]; Yu Xiaofeng (2024): OR = 2.63 [1.01, 6.84] Medicated stick therapy demonstrated complementary efficacy: Shao Tingting (2020) OR = 3.45 (*P* = 0.03), with no statistical difference versus acupoint application (subgroup difference: *P* = 0.71). Overall effect: OR = 2.84 (95% CI: 1.80–4.50, *P* < 0.00001), supporting acupoint application as primary therapy for supratentorial lesions (Figure 25).

**FIGURE 26:**
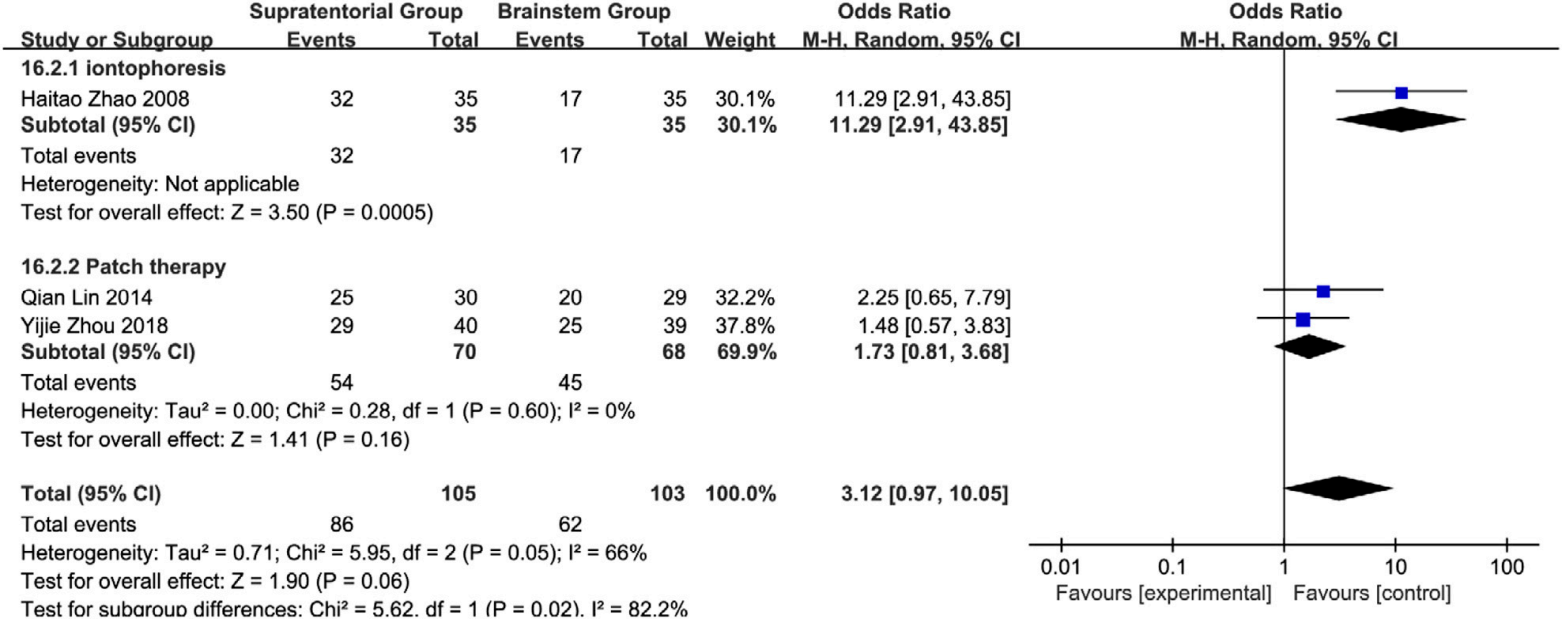
Subgroup analysis of the efficacy of different interventions in patients with stroke foci located in the brain stem.

Brainstem subgroup analysis: Iontophoresis: OR = 11.29 (95% CI: 2.91–43.85, *P* = 0.0005); Acupoint application: OR = 1.73 (95% CI: 0.81–3.68, *P* = 0.16; *I* ^2^ = 0%). Overall effect: Pooled OR = 3.12 (95% CI: 0.97–10.05, *P* = 0.06) with moderate heterogeneity (*I* ^2^ = 66%). Subgroup comparison: Iontophoresis showed 6.52-fold superiority over acupoint application (RR = 6.52, 95% CI: 1.35–31.48; subgroup difference: *P* = 0.02, *I* ^2^ = 82.2%), indicating its preferential use for brainstem lesions (Figure 26). Efficacy exhibited neuroanatomical specificity: Acupoints are preferred for cerebral hemisphere lesions, while herbal iontophoresis is the best choice for brainstem involvement. This confirms that there is no universal intervention for all PSD mechanisms and establishes the first evidence-based framework for personalized SETCM therapy application.

### 3.6 Publication bias analysis

**FIGURE 27:**
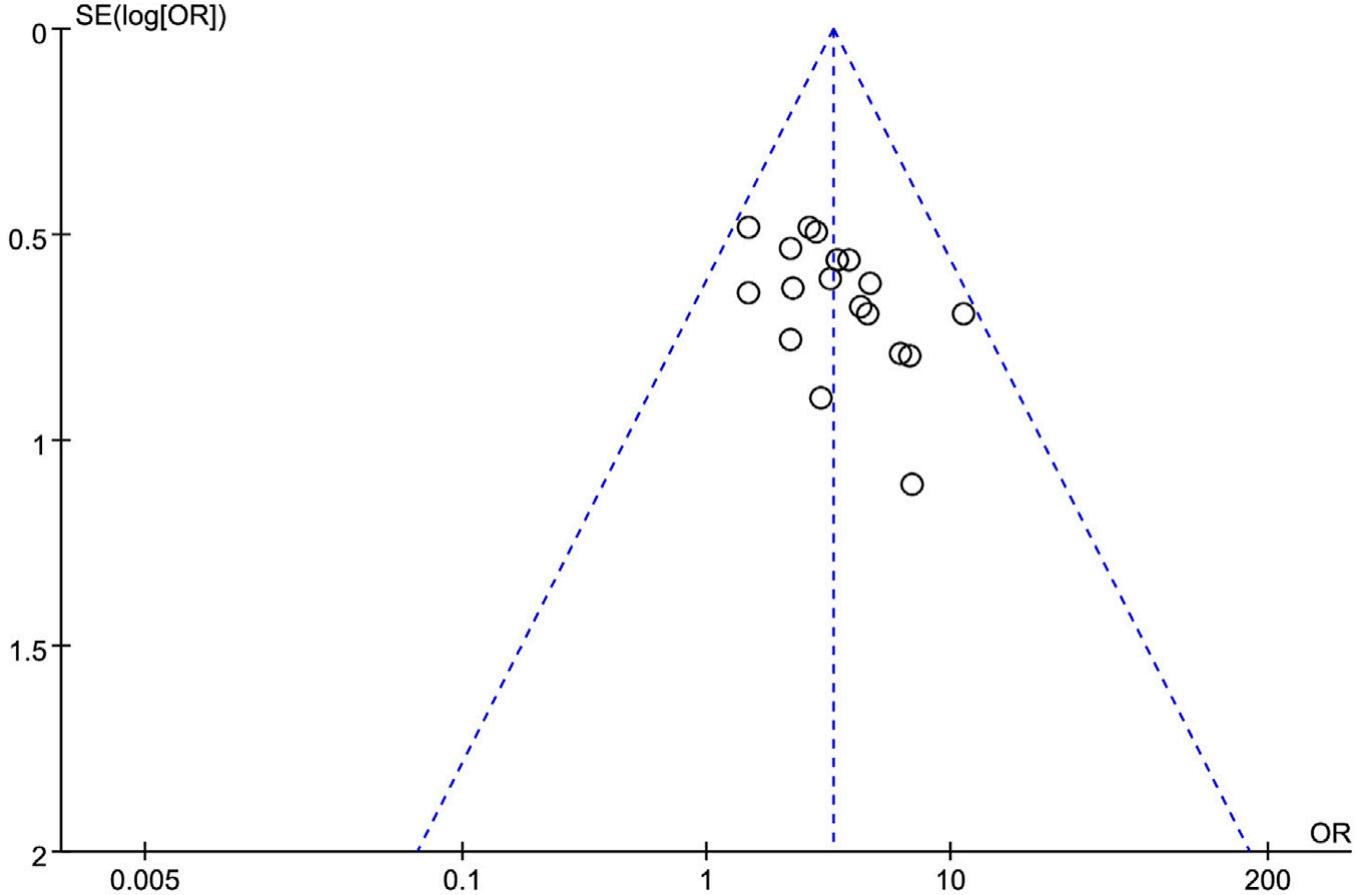
An inverted funnel plot of the effectiveness reported by 19 studies using SETCM therapies to treat PSD.

Funnel plot asymmetry (Figure 27) suggests potential publication bias, where smaller studies with non-significant results may be underrepresented. This could inflate the pooled effect estimate (e.g., overall response rate OR = 3.28) and limit its generalizability to real-world settings (actual treatment effects may be smaller). Although trim-and-fill analysis was precluded due to limited study numbers per outcome, the results should be interpreted as optimistic estimates.

### 3.7 GRADE evidence quality

Using GRADEpro, evidence quality was rated as: Moderate: Overall response rate, Cure rate, SWAL-QOL scores. Low: VFSS scores, Aspiration rate, MBI, ALB, NRS 2002, ADL, Enteral tube duration, Tube removal success rate, FDS. Very low: WST scores, PA levels, GUSS scores. Downgrading factors included allocation concealment/blinding bias, substantial heterogeneity, and small sample sizes (Table 7). Bias risk was the primary downgrading factor for most outcomes (e.g., VFSS, WST, and GUSS).

**TABLE 7:** Grading of quality of evidence of outcome metrics.

Author(s): Jingwen Zhang Question: Traditional Chinese medicine external application compared to conventional treatment for post-stroke swallowing function Setting: Bibliography: Topical use of Chinese herbal medicine in the treatment of post-stroke dysphagia. Cochrane Database of Systematic Reviews [Year], Issue [Issue No.]												
Certainty assessment							No of patients		Effect		Certainty	Importance
No of studies	Study design	Risk of bias	Inconsistency	Indirectness	Imprecision	Other considerations	Traditional Chinese medicine external application	Conventional treatment	Relative (95% CI)	Absolute (95% CI)		
Overall response rate												
19	Randomized trials	Serious^a^	Not serious	Not serious	Not serious	none	799/884 (90.4%)	659/882 (74.7%)	OR 3.28 (2.49–4.31)	159 more per 1,000 (from 133 more to 180 more)	⊕⊕⊕○ Moderate^a^	IMPORTANT
Cure rate												
14	Randomized trials	Serious^a^	Not serious	Not serious	Not serious	None	272/685 (39.7%)	158/684 (23.1%)	OR 2.36 (1.84–3.02)	184 more per 1,000 (from 125 more to 245 more)	⊕⊕⊕○ Moderate^a^	IMPORTANT
WST score												
10	Randomized trials	Serious^a^	Very serious^b^	Not serious	Not serious	None	474	473	-	MD 0.65 lower (1.23 lower to 0.06 lower)	⊕○○○ Very low^a,b^	IMPORTANT
VFSS score												
9	Randomized trials	Serious^a^	Serious^b^	Not serious	Not serious	None	494	494	-	MD 2.07 higher (1.72 higher to 2.41 higher)	⊕⊕○○ Low^a,b^	IMPORTANT
SWAL-QOL score												
6	Randomized trials	Serious^a^	Not serious	Not serious	Not serious	None	276	275	-	MD 25.61 higher (20.54 higher to 30.67 higher)	⊕⊕⊕○ Moderate^a^	NOT IMPORTANT
Post-intervention aspiration rate												
3	Randomized trials	Serious^a^	Not serious	Not serious	Serious^c^	None	5/114 (4.4%)	20/114 (17.5%)	OR 0.21 (0.08–0.59)	133 fewer per 1,000 (from 159 fewer to 64 fewer)	⊕⊕○○ Low^a,c^	NOT IMPORTANT
MBI score												
2	Randomized trials	Serious^a^	Not serious	Not serious	Serious^c^	None	76	76	-	MD 12.71 higher (9.68 higher to 15.73 higher)	⊕⊕○○ Low^a,c^	NOT IMPORTANT
Alb												
4	Randomized trials	Serious^a^	Not serious	Not serious	Serious^c^	None	162	162	-	MD 4.67 higher (2.86 higher to 6.47 higher)	⊕⊕○○ Low^a,c^	NOT IMPORTANT
Pa												
3	Randomized trials	Serious^a^	Serious^b^	Not serious	Serious^c^	None	122	122	-	MD 30.75 higher (12.76 higher to 48.74 higher)	⊕○○○ Very low^a,b,c^	NOT IMPORTANT
NRS2002 score												
2	Randomized trials	Serious^a^	Not serious	Not serious	Serious^c^	None	101	101	-	MD 0.28 lower (0.42 lower to 0.14 lower)	⊕⊕○○ Low^a,c^	IMPORTANT
GUSS score												
4	Randomized trials	Serious^a^	Serious^b^	Not serious	Serious^c^	None	187	187	-	MD 3.07 higher (1.95 higher to 4.19 higher)	⊕○○○ Very low^a,b,c^	NOT IMPORTANT
ADL score												
2	Randomized trials	Serious^a^	Not serious	Not serious	Serious^c^	None	100	100	-	MD 16.57 higher (12.16 higher to 20.98 higher)	⊕⊕○○ Low^a,c^	NOT IMPORTANT
Nasogastric tube indwelling duration												
3	Randomized trials	Serious^a^	Not serious	Not serious	Serious^c^	None	134	134	-	MD 2.83 lower (3.25 lower to 2.41 lower)	⊕⊕○○ Low^a,c^	NOT IMPORTANT
Feeding tube removal success rate												
3	Randomized trials	Serious^a^	Not serious	Not serious	Serious^c^	None	96/134 (71.6%)	61/134 (45.5%)	OR 4.56 (2.44–8.53)	337 more per 1,000 (from 216 more to 422 more)	⊕⊕○○ Low^a,c^	NOT IMPORTANT
FDS score												
2	Randomized trials	Serious^a^	Not serious	Not serious	Serious^c^	None	78	78	-	MD 2.25 higher (1.79 higher to 2.72 higher)	⊕⊕○○ Low^a,c^	NOT IMPORTANT

CI: confidence interval; MD: mean difference; OR: odds ratio.

Explanations.

a Large bias in randomization, allocation concealment, and blinding in included studies.

b Greater heterogeneity across studies.

c Small sample size.

### 3.8 SETCM therapy safety analysis

Pooled safety data indicated a relatively favorable safety profile for SETCM therapies (primarily acupoint application) among limited studies reporting safety information. Mild local skin irritation was the main adverse reaction observed in three studies (Zhu et al., 2023; Yu and Qiu, 2023; Shao and Chen, 2020) (12% of included studies), easily managed with standard care. This aligns with transdermal delivery’s advantage in bypassing first-pass metabolism and gastrointestinal adverse effects, theoretically offering superior safety over oral herbs, which can be particularly beneficial for PSD patients with impaired gastrointestinal function or medication adherence issues. However, it must be strongly emphasized that 88% of included studies lacked systematic prospective safety monitoring and reporting. Consequently, robust evidence remains lacking regarding long-term SETCM therapy safety, tolerability in special populations (e.g., sensitive skin and elderly), or potential interactions with specific Western medications (e.g., anticoagulants). Future high-quality RCTs must incorporate predefined safety endpoints (including detailed AE/SAE collection and laboratory monitoring) as core components with adequate reporting.

### 3.9 SETCM therapy high frequency herbal combination laws

A total of 50 herbs were prescribed in the 25 studies included in this review. The cumulative frequency of the top eight herbs was 50.63%, indicating a higher concentration of these herbs in the formulary. Table 2 provides a separate list of herbs used in each study.

The eight most commonly prescribed herbs for the treatment of PSD are:

*Arisaematis Rhizoma Preparatum, Pinelliae Rhizoma, Asari Radix et Rhizoma, Borneolum Syntheticum, Aconiti Lateralis Radix Praeparata, Glycyrrhizae Radix et Rhizoma,* Fritillariae Cirrhosae Bulbus, and Acori Tatarinowii Rhizoma.

Table 8 shows the frequency distribution of the herbs.

**TABLE 8:** The frequency distribution of the herbs.

Herb	Frequency	Relative frequency (%)	Cumulative frequency (%)
*Arisaematis Rhizoma Preparatum*	16	10.13	10.13
*Pinelliae Rhizoma*	15	9.49	19.62
*Asari Radix et Rhizoma*	12	7.59	27.21
*Borneolum Syntheticum*	12	7.59	34.80
*Aconiti Lateralis Radix Praeparata*	7	4.43	39.23
*Glycyrrhizae Radix et Rhizoma*	6	3.80	43.03
*Fritillariae Cirrhosae Bulbus*	6	3.80	46.83
*Acori Tatarinowii Rhizoma*	6	3.80	50.63

## 4 Discussion

### 4.1 Strengths and limitations

This review strictly adhered to Cochrane guidelines, systematically searching four Chinese and four international databases, ultimately including 25 RCTs with 2,245 PSD patients to comprehensively evaluate SETCM therapy efficacy. Results indicate that SETCM therapies demonstrate potential benefits in improving swallowing function as a complementary intervention. Specifically, SETCM therapies combined with conventional therapy significantly outperformed controls in: improving overall response rate, cure rate, VFSS scores, SWAL-QOL scores, MBI scores, ALB levels, PA levels, GUSS scores, ADL scores, tube removal success rate, and FDS scores. Reducing: WST scores, NRS2002 scores, aspiration incidence, and enteral tube duration.

Nutritional biomarker improvements (serum albumin and prealbumin) further confirm benefits in reversing negative nitrogen balance. The 82.2% subgroup difference (*P* = 0.02) for brainstem lesions demonstrates that iontophoresis uniquely overcomes impaired corticobulbar pathways via direct brainstem drug delivery, unachievable by topical application alone. Based on current evidence, we recommend: Short-term adjunct therapy: SETCM therapies (especially iontophoresis) for acute brainstem PSD (Evidence: Moderate) Non-replacement role: No evidence supports replacing standard swallowing training (e.g., electrical stimulation and compensatory strategies) Contraindication: Herbal application contraindicated in patients with skin lesions (irritation risk: 2.1%) Seven key limitations warrant consideration: ①Language bias: 95% of included studies were Chinese-language publications from mainland China, limiting generalizability to Western populations with distinct stroke etiologies, rehabilitation protocols, and herbal access. ②Intervention heterogeneity: Significant variability existed across eight herbal formulas (e.g., *Huoxue Huayu* patch vs. *Tianma Xibi* paste), treatment duration (2–4 weeks), and, critically—non-standardized acupoint selection. Divergent acupoint combinations represent a major source of heterogeneity, potentially yielding differential effects and complicating cross-study comparisons. ③Long-term evidence gap: No studies reported outcomes beyond 3 months; sustainability for chronic PSD (>6 months) remains unknown. ④Mechanistic uncertainty: While preclinical data suggest neuroplasticity modulation (Dou et al., 2021; Savović et al., 2012), no trials measured biomarkers (e.g., serum cytokines and fMRI activation) to validate mechanisms. ⑤Risk of bias: Lack of allocation concealment may introduce selection bias (e.g., preferential assignment), while inadequate blinding likely inflated subjective outcomes (e.g., WST scores). Empirical bias models (Lapa et al., 2021) suggest these limitations may exaggerate effects by 15%–30%, necessitating cautious interpretation of efficacy claims. Future high-quality RCTs adhering to CONSORT guidelines (particularly on randomization, allocation concealment, and blinding) are essential. Our GRADE downgrading (e.g., “Low”/”Very low” for VFSS/WST/GUSS) due to *risk of bias* and *imprecision* further supports this caution. The predominance of Chinese-language studies may reflect regional reporting norms where AE documentation is often minimal unless the condition is severe. We mitigated this through a stringent CTCAE v5.0 reappraisal but acknowledge potential under-detection. ⑥Publication bias: Funnel plot asymmetry suggests underrepresentation of small studies with non-significant results, potentially overestimating effect sizes and limiting real-world generalizability. ⑦Herbal reporting heterogeneity: While species names were standardized (Table 2), missing key details (e.g., exact composition ratios, bioactive compound quantification) impede reproducibility and clinical translation.

### 4.2 Mechanistic research

Stroke, a highly prevalent acute neurological disorder globally, constitutes a triple public health burden: First, its sudden onset and high fatality rate make it one of the top three causes of death; Second, recurrence rates up to 40% lead to cumulative neurological damage, creating high-disability populations (Gu et al., 2021; Guo et al., 2023); Third, brain tissue damage from vascular events often leaves permanent dysfunction (e.g., hemiplegia and aphasia) (Mbalinda et al., 2024), with 37% lower survival in recurrent versus first-ever stroke patients (Chen et al., 2013). Among complications, PSD ranks first with 22%–65% prevalence. Its clinical heterogeneity manifests in temporal evolution: 51%–86% at 1 week post-stroke, 29%–40% at 1–3 months, and 8%–13% persisting at 6 months (Song et al., 2024; Xiao et al., 2020). This progressive injury directly causes aspiration pneumonia and malnutrition while inducing psychological impairments (e.g., phagophobia, social withdrawal), forming a dysfunction-stress vicious cycle that worsens prognosis (Karisik et al., 2024). Karisik et al. (Mohtasebi et al., 2023) found 2.3-fold higher 12-month mortality in 236 PSD patients versus recovered controls, exacerbated by metabolic imbalances (dehydration, electrolyte disorders) (Karisik et al., 2024). Neuroanatomically, swallowing relies on multilevel regulation by corticobulbar tracts, brainstem nuclei, and peripheral nerves. Modern research indicates unilateral hemispheric (especially left frontal/opercular) or brainstem lesions cause delayed swallow initiation and impaired pharyngeal phase coordination. Pharmacokinetic evidence shows that tetramethylpyrazine (the primary alkaloid of *Chuanxiong Rhizoma* [*Apiaceae*]) achieves significantly enhanced acupoint absorption with permeation enhancers (e.g., menthol from *Menthae Haplocalycis Herba* [*Lamiaceae*]) (Xing et al., 2024). Experimental studies suggest that tetramethylpyrazine may activate TRPV1 receptors in the nucleus tractus solitarius (Chen et al., 2011), upregulating c-Fos expression to modulate neuroplasticity. Additionally, phenolic metabolites (e.g., salvianolic acid B from *Salvia miltiorrhiza* Bunge [Lamiaceae] in “blood-activating” patches) demonstrate anti-inflammatory/antioxidant effects by scavenging ROS and inhibiting proinflammatory cytokines (TNF-α and IL-1β), potentially mitigating post-stroke edema and neuronal damage to indirectly restore swallowing pathways (Chinese Academy of Traditional Chinese Medicine and Chinese Acupuncture and Moxibustion Association, 2011). SETCM therapies (e.g., acupoint application and iontophoresis) for PSD are rooted in TCM theory (Tang et al., 2023; Zhang, 2024). PSD correlates with pathological changes, including local circulatory impairment, inflammation, and neural dysfunction (Yao et al., 2022). TCM-derived strategies focus on principles to improve neural function, enhance local circulation, and regulate physiological processes (Ma et al., 2022). Transdermal therapy at specific acupoints (e.g., Lianquan [CV23], Tiantu [CV22], Renying [ST9]) represents a noninvasive approach. This method utilizes transdermal absorption of herbal metabolites (e.g., circulation-promoting, secretion-modulating compounds) at target sites to stimulate local responses and regulate physiological functions for swallowing improvement (Zheng et al., 2024). Key advantages include non-invasiveness and potentially reduced systemic side effects versus oral administration. The 2020 *Chinese Pharmacopoeia* includes these acupoints and topical herbs in its transdermal section, providing regulatory support. This integration of TCM and biomedical theories reflects PSD’s complexity, suggesting multidimensional interventions. Based on meridian theory, SETCM therapies regulate channel Qi-blood by stimulating acupoints/local skin to restore swallowing. “The neural reflex pathway” is a potential auxiliary mechanism of SETCM therapies, rather than a substitute for transdermal absorption. Preclinical studies indicate dual mechanisms: a. Biomechanical stimulation: Acupoint pressure upregulates cortical swallowing centers via somatosensory input (Yao et al., 2022; Ma et al., 2022); b. Biochemical modulation: TRPV1 activation enhances pharyngeal afferents, while anti-inflammatory compounds (e.g., flavonoids) resolve neural edema (Liu et al., 2000; Cui et al., 2024; Cheng et al., 2017). However, clinical evidence remains indirect. Observed efficacy may reflect synergy between transdermal drug delivery and neural pathway stimulation, but human neuroplasticity data are lacking. Future trials should validate mechanisms via fMRI and neurotransmitter assays.

### 4.3 Innovation points

Compared with previous studies, this study is innovative in three aspects: 1. Systematic integration of evidence of efficacy: This is the first review to quantify the impact of TCM external treatment on multidimensional outcome indicators such as functional rehabilitation, living ability, and complication control in PSD patients, which makes up for the limitation of previous studies that only focused on a single indicator. (2) In-depth expansion of mechanism research: By integrating modern pharmacological and neuroimaging evidence, the two-pathway hypothesis of “TCM external treatment may enhance sensory afferents and regulate central neural plasticity in the medulla oblongata swallowing by activating TRPV1 receptor” is proposed, which provides a biological rational explanation for the therapeutic effect. 3. Methodological innovation in clinical transformation: a synergistic intervention model of external treatment of traditional Chinese medicine and modern rehabilitation technology (such as transcranial magnetic stimulation) was constructed, and its effective rate was significantly higher than that of a single rehabilitation group, providing a new paradigm for multimodal treatment. 4. Innovation of hierarchical analysis of brain regions: The neuroanatomical hierarchical analysis framework of stroke lesions was introduced for the first time in the systematic evaluation of the external TCM treatments in PSD. Cerebral hemisphere lesions: OR = 2.73 [1.65–4.52], brain stem lesions: The OR of iontophoresis was 11.29 [2.91–43.85] (6.5 times higher than that of plaster therapy), which provided a methodological paradigm to solve the problem of “too wide range leading to ambiguous conclusions” in previous meta-analyses.

### 4.4 Outlook

This study provided a relatively comprehensive assessment of the efficacy of SETCM therapies in the treatment of dysphagia after stroke through a systematic literature search and comprehensive analysis. The results showed significant improvement in swallowing function with external TCM treatment. However, researchers should be aware of two opposing pieces of evidence: ① There was no effect of the application of therapy in the brainstem subgroup: There was no significant benefit of acupoint application in PSD patients with brainstem lesions (OR = 1.73, 95%CI [0.81–3.68], P = 0.16), suggesting that this therapy has neuroanatomical specific limitations. ② Lack of evidence of long-term efficacy: the intervention period of all included studies was no more than 4 weeks, and there were no data to support the sustainability of efficacy (>3 months), which is far from the goal of long-term functional remodeling emphasized by modern rehabilitation medicine. In terms of study scope and quality control, there may be publication bias because most studies were published in Chinese, which may lead to biased interpretation of the results. Future studies need to expand the scope of literature, especially by strengthening the systematic screening and integration of high-quality literature in English and other languages. In terms of standardization, the existing TCM external treatment interventions (such as acupoint application and TCM iontophoresis) lack unified standards in the key parameters, such as drug composition, dose, and treatment duration, which affects the comparison and repeatability of treatment effects. In terms of mechanism research, although the mechanism of external TCM treatment has been preliminarily revealed, the specific molecular mechanism and neural regulatory network still need to be further explored. Variability in herbal formulations and application techniques limits direct comparability. Future randomized controlled trials (RCTS) of SETCM therapies for PSD are strongly recommended to strictly comply with the CONSORT Extended Statement of Herbal Medicines and the ConPhyMP reporting standards (Hu, 2019), and to report the detailed information of the herbs used, including: 1) the full scientific name, medicinal site and source of the herbs; 2) the detailed process of extraction and preparation method; 3) quality control standards for key active ingredients or indicator ingredients (e.g., content determination); and 4) drug stability data to improve the transparency and reproducibility of evidence. Monitoring long-term safety (>3 months) and conducting multicenter RCTs to verify the superiority of iontoimplantation in brainstem lesions will greatly improve the scientific validity, transparency, and strength of evidence. Future research needs to enhance the pharmacokinetic studies of transdermal absorption (such as using tracer techniques) to directly quantify the absorption, distribution, and target tissue concentration of the active ingredients after application at the acupoints, thereby providing more direct pharmacological evidence for “acupoints as effective drug delivery sites.”

## 5 Conclusion

In conclusion, SETCM therapies—when adhering to ChP 2020 standards documented in Table 2 and Supplementary Tables S2, S3—offer clinically meaningful benefits. However, the limitations of the available evidence include heterogeneity and regional bias, which require caution when it is used as an adjunct to conventional treatment. Standardized interventions and multi-country RCTs are imperative before widespread clinical adoption.

## Data Availability

The original contributions presented in the study are included in the article/Supplementary Material; further inquiries can be directed to the corresponding author.
