# Supplementary material for: Systematic review and meta-analysis of specific external Chinese herbal medicines for post-stroke dysphagia: efficacy and clinical implications
Source: Front Pharmacol. 2025 Sep 1;16:1635090. doi: 10.3389/fphar.2025.1635090 (PMC12434097; doi:10.3389/fphar.2025.1635090)
Supplement: Supplementary file 1 [file Table1.docx]

The English search was conducted using PubMed as an example:

| #1 | ((("Stroke"[Mesh]) OR (((((((((((((((((((((((((((Cerebrovascular Accident[Title/Abstract]) OR (Cerebrovascular Accidents[Title/Abstract])) OR (Cerebral Stroke[Title/Abstract])) OR (Cerebral Strokes[Title/Abstract])) OR (Stroke, Cerebral[Title/Abstract])) OR (Strokes, Cerebral[Title/Abstract])) OR (Cerebrovascular Apoplexy[Title/Abstract])) OR (Apoplexy, Cerebrovascular[Title/Abstract])) OR (Vascular Accident, Brain[Title/Abstract])) OR (Brain Vascular Accident[Title/Abstract])) OR (Brain Vascular Accidents[Title/Abstract])) OR (Vascular Accidents, Brain[Title/Abstract])) OR (Cerebrovascular Stroke[Title/Abstract])) OR (Cerebrovascular Strokes[Title/Abstract])) OR (Stroke, Cerebrovascular[Title/Abstract])) OR (Strokes, Cerebrovascular[Title/Abstract])) OR (Apoplexy[Title/Abstract])) OR (CVA (Cerebrovascular Accident[Title/Abstract]))) OR (CVAs (Cerebrovascular Accident[Title/Abstract]))) OR (Stroke, Acute[Title/Abstract])) OR (Acute Stroke[Title/Abstract])) OR (Acute Strokes[Title/Abstract])) OR (Strokes, Acute[Title/Abstract])) OR (Cerebrovascular Accident, Acute[Title/Abstract])) OR (Acute Cerebrovascular Accident[Title/Abstract])) OR (Acute Cerebrovascular Accidents[Title/Abstract])) OR (Cerebrovascular Accidents, Acute[Title/Abstract]))) |
| --- | --- |
| #2 | (((("Deglutition Disorders"[Mesh]) OR (((((((((((Deglutition Disorder[Title/Abstract]) OR (Disorders, Deglutition[Title/Abstract])) OR (Dysphagia[Title/Abstract])) OR (Swallowing Disorder[Title/Abstract])) OR (Swallowing Disorders[Title/Abstract])) OR (Oropharyngeal Dysphagia[Title/Abstract])) OR (Dysphagia, Oropharyngeal[Title/Abstract])) OR (Esophageal Dysphagia[Title/Abstract])) OR (Dysphagia, Esophageal[Title/Abstract])) OR (deglutition difficulty[Title/Abstract])) OR (swallowing difficulty[Title/Abstract]))) |
| #3 | (("Medicine, Chinese Traditional"[Mesh]) OR ((((((((((((((Zhong Yi Xue[Title/Abstract]) OR (Chung I Hsueh[Title/Abstract])) OR (Hsueh, Chung I[Title/Abstract])) OR (Traditional Medicine, Chinese[Title/Abstract])) OR (Chinese Traditional Medicine[Title/Abstract])) OR (Traditional Chinese Medicine[Title/Abstract])) OR (Chinese Medicine, Traditional[Title/Abstract])) OR (Traditional Tongue Diagnosis[Title/Abstract])) OR (Tongue Diagnoses, Traditional[Title/Abstract])) OR (Tongue Diagnosis, Traditional[Title/Abstract])) OR (Traditional Tongue Diagnoses[Title/Abstract])) OR (Traditional Tongue Assessment[Title/Abstract])) OR (Tongue Assessment, Traditional[Title/Abstract])) OR (Traditional Tongue Assessments[Title/Abstract])))) |
| #4 | (clinicaltrial[Filter] OR randomizedcontrolledtrial[Filter])) |
| #5 | (randomized controlled trial[Publication Type] OR randomized[Title/Abstract] OR placebo[Title/Abstract]) |
| #6 | #1AND#2AND#3AND#4AND#5 |
